# Vaccine-induced inflammation and inflammatory monocytes promote CD4^+^ T cell-dependent immunity against murine salmonellosis

**DOI:** 10.1371/journal.ppat.1011666

**Published:** 2023-09-21

**Authors:** Nancy Wang, Timothy A. Scott, Andreas Kupz, Meghanashree M. Shreenivas, Newton G. Peres, Dianna M. Hocking, Chenying Yang, Leila Jebeli, Lynette Beattie, Joanna R. Groom, Thomas P. Pierce, Linda M. Wakim, Sammy Bedoui, Richard A. Strugnell

**Affiliations:** 1 Department of Microbiology and Immunology, The University of Melbourne, at Peter Doherty Institute for Infection and Immunity, Melbourne, Victoria, Australia; 2 Walter and Eliza Hall Institute of Medical Research, Parkville, Victoria, Australia; 3 Department of Medical Biology, The University of Melbourne, Parkville, Victoria, Australia; 4 Ludwig Institute for Cancer Research, Melbourne-Parkville Branch, Parkville, Victoria, Australia; University of California Davis School of Medicine, UNITED STATES

## Abstract

Prior infection can generate protective immunity against subsequent infection, although the efficacy of such immunity can vary considerably. Live-attenuated vaccines (LAVs) are one of the most effective methods for mimicking this natural process, and analysis of their efficacy has proven instrumental in the identification of protective immune mechanisms. Here, we address the question of what makes a LAV efficacious by characterising immune responses to a LAV, termed TAS2010, which is highly protective (80–90%) against lethal murine salmonellosis, in comparison with a moderately protective (40–50%) LAV, BRD509. Mice vaccinated with TAS2010 developed immunity systemically and were protected against gut-associated virulent infection in a CD4^+^ T cell-dependent manner. TAS2010-vaccinated mice showed increased activation of Th1 responses compared with their BRD509-vaccinated counterparts, leading to increased Th1 memory populations in both lymphoid and non-lymphoid organs. The optimal development of Th1-driven immunity was closely correlated with the activation of CD11b^+^Ly6G^neg^Ly6C^hi^ inflammatory monocytes (IMs), the activation of which can be modulated proportionally by bacterial load *in vivo*. Upon vaccination with the LAV, IMs expressed T cell chemoattractant CXCL9 that attracted CD4^+^ T cells to the foci of infection, where IMs also served as a potent source of antigen presentation and Th1-promoting cytokine IL-12. The expression of MHC-II in IMs was rapidly upregulated following vaccination and then maintained at an elevated level in immune mice, suggesting IMs may have a role in sustained antigen stimulation. Our findings present a longitudinal analysis of CD4^+^ T cell development post-vaccination with an intracellular bacterial LAV, and highlight the benefit of inflammation in the development of Th1 immunity. Future studies focusing on the induction of IMs may reveal key strategies for improving vaccine-induced T cell immunity.

## Author summary

*Salmonella enterica* infections exemplify the immunological challenges posed by intracellular bacterial pathogens, for which there are often limited or no effective vaccines and antimicrobial resistance is rapidly on the rise. A common signature among these infections is a strong dependence on CD4^+^ T cell responses for host immunity, although how such responses can be effectively induced in a vaccine setting remains a key challenge. Using two live-attenuated vaccines that offer distinct levels of protection against lethal salmonellosis in a murine model, we investigated what properties of vaccine-induced immune responses can be targeted for improving vaccine efficacy. Our data show that the longevity of activated CD4^+^ T cells in lymphoid and non-lymphoid organs is closely linked with vaccine efficacy. At the cellular level, we have shown that CD11b^+^Ly6G^neg^Ly6C^hi^ inflammatory monocytes play an important role in stimulating antigen-specific CD4^+^ T cells through antigen presentation mechanisms, as well as the production of CXCL9 and IL-12. Since our data suggest that acute inflammation is beneficial for optimising vaccine-induced T cell immunity, considerations should be given to preserving the targets of inflammatory signalling pathways as a means for improving vaccine efficacy in future development.

## Introduction

The species *Salmonella enterica* encompasses closely-related facultative intracellular bacterial pathogens that transmit via the faecal-oral route, but the resulting disease presentation and severity differ considerably [1]. These presentations include enteric fever (including typhoid fever and the less common paratyphoid fever), caused by human-restricted serovars Typhi and Paratyphi [2]; and non-typhoidal salmonellosis (NTS), predominantly caused by broad-host-range serovars Typhimurium and Enteritidis, for which disease presentation may range from self-limiting gastroenteritis to life-threatening bacteraemia known as invasive NTS (iNTS) [3]. For both enteric fever and iNTS, a key bottleneck event in disease progression is bacterial dissemination from the gastrointestinal (GI) tract, escalating a localised gut infection to systemic disease with severe complications including fever, delirium and intestinal perforations [4,5]. For typhoid fever, a live-attenuated vaccine, Ty21a, offers moderate protection, and the recent rollout of a protein-conjugated capsular Vi antigen subunit vaccine (TCV) has shown improved (albeit still incomplete) protection than Vi capsular polysaccharide vaccines [6]. On the other hand, no human vaccines are currently licensed for non-typhoidal serovars, which lack the Vi capsular antigen [7], and T cell-based mechanisms are thought to represent a strong target for vaccine development [8].

As with many intracellular bacterial pathogens, CD4^+^ T cells are essential for controlling *S*. *enterica* infection. Individuals with declining CD4^+^ T cell numbers due to HIV are highly susceptible to iNTS, and the case-fatality rate for HIV/iNTS co-infection can be as high as 20–25% in sub-Saharan Africa [3]. Genetic deficiencies for Th1 effector molecules (e.g. IFN-γ), Th1-inducing cytokines (e.g. IL-12) or their receptors are associated with drastically increased susceptibility to *S*. *enterica* [9]. This strong dependence on Th1-driven immunity is closely recapitulated in murine infection models of *S*. *enterica* serovar Typhimurium (*S*. Typhimurium). In mice with the C57BL/6 genetic background, infection with wild-type *S*. Typhimurium leads to rapid lethality. Attenuated *S*. Typhimurium strains, most notably those with mutations in the pre-chorismate biosynthetic pathway (e.g. *ΔaroA* mutants SL3261 and BRD509), show much reduced growth *in vivo* and are frequently used to model human primary *S*. *enterica* infection [10,11]. The systemic control and clearance of primary infection with attenuated *S*. Typhimurium requires Th1-polarised CD4^+^ T cells [11–17], while a Th17 population contributes to controlling extracellular bacteria from breaching the gastrointestinal barrier [18–20]. Mice that have cleared primary infection with attenuated *S*. Typhimurium become immune and require CD4^+^ T cells for protection against challenge with virulent *S*. Typhimurium [11,17,21,22], while CD8^+^ T cells [13,16,23] and B cell responses [24–26] likely make additional contributions to overall immunity. This vaccine/challenge model system has proven valuable for evaluating the efficacy of different vaccine constructs as well as for deconvoluting the cell types and mechanisms that contribute to protective immunity [27,28].

We have previously generated a *S*. Typhimurium mutant that is deficient in central carbon metabolism, denoted TAS2010 (Δ*pfkA*Δ*pfkB*Δ*edd*), which as a live-attenuated vaccine (LAV) confers superior protection against lethal challenge compared with the ‘benchmark’ LAV strain, BRD509 (Δ*aroA*) [29]. In the present study, we compared post-vaccination immune responses to these two LAVs in an effort to identify mechanisms by which LAVs induce high-quality, protective immunity. We contrasted the kinetics of post-vaccination CD4^+^ T cell responses from priming to the memory phase, and conducted a detailed analysis of the inflammatory environment that developed in proportion to the antigen dose. Our results point to CD11b^+^Ly6G^neg^Ly6C^hi^ inflammatory monocytes as an important link between innate immune activation and T cell-mediated immunity, and suggest that inflammatory responses to vaccination are necessary for achieving optimal T cell induction.

## Results

### Enhanced protective immunity develops systemically after infection with *S*. Typhimurium LAV strain TAS2010

**Fig 1:**
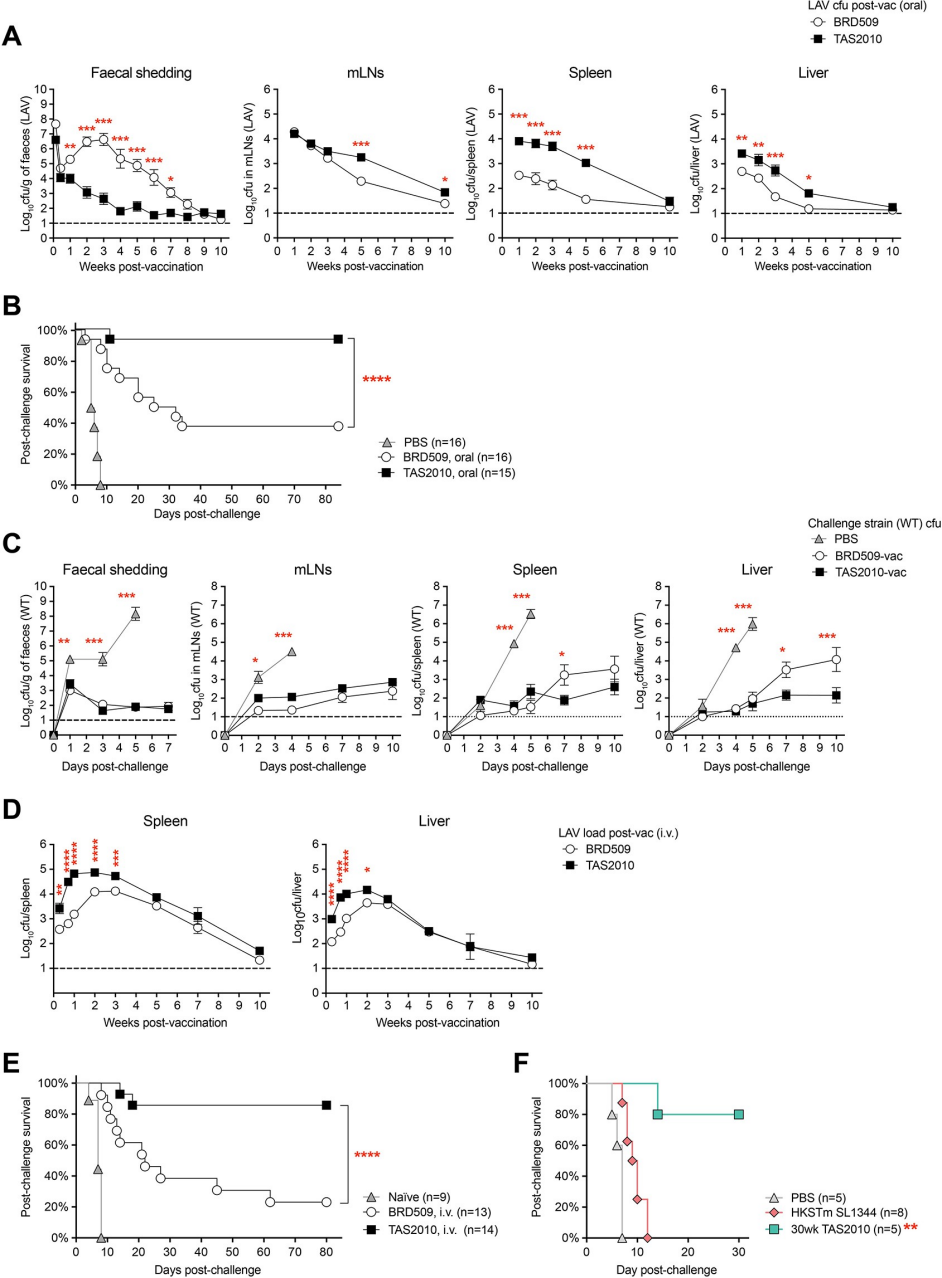
The *S*. Typhimurium live-attenuated vaccine (LAV) strain TAS2010 shows enhanced growth in tissues and confers potent protection against challenge. **A-C)** Wild-type C57BL/6 mice were given an oral gavage of 5×10^9^cfu *S*. Typhimurium LAV strain BRD509 (Δ*aroA*) or TAS2010 (Δ*pfkA*Δ*pfkB*Δ*edd*) as the vaccine (vac), or an oral gavage of PBS only (PBS). At week 10–12 post-vaccination, mice were challenged with an oral gavage of 10^7^cfu wild-type *S*. Typhimurium SL1344. A) The bacterial load of *S*. Typhimurium LAV strains was determined in the mesenteric lymph nodes (mLNs) (n = 9–11), faeces (n = 10–35), spleen (n = 9–17) and liver (n = 9–17) at the indicated time points post-vaccination. B) Shown is the percentage of mice remaining protected at the indicated time points post-challenge. C) The growth of wild-type *S*. Typhimurium SL1344 in challenged mice was determined in the mLNs (n = 8–10), faeces (n = 10), spleen (n = 6–12) and liver (n = 6–12) at the indicated time points post-challenge. Symbols indicate geometric mean of bacterial load ± SEM, with data pooled from 2–4 independent experiments. Two-way ANOVA with Bonferroni’s post-tests were used for comparing the three groups, and asterisks indicate significant differences between the indicated group and the TAS2010-vaccinated group. **D, E)** Wild-type C57BL/6 mice were i.v. injected with 200cfu TAS2010 or BRD509. D) The bacterial load was determined in the spleen and liver at the indicated time points post-vaccination. Symbols indicate geometric mean of bacterial load ± SEM (n = 5–23), data at each time point are pooled from 2–4 independent experiments. E) At week 10–12 post-vaccination, naïve or vaccinated mice were challenged with an oral gavage of 10^7^cfu wild-type *S*. Typhimurium SL1344. Shown is the percentage of mice remaining protected at the indicated time points post-challenge. **F)** Wild-type C57BL/6 mice were i.v. vaccinated with either a single dose of 200cfu TAS2010 and challenged 30 weeks later, or four doses of 5×10^7^cfu heat-killed *S*. Typhimurium SL1344 (HKSTm) or PBS at day 0, 3, 7 and 14, then challenged at day 28. Challenge was given as 10^7^cfu wild-type *S*. Typhimurium SL1344 by oral gavage. Shown is the percentage of mice remaining protected at the indicated time points post-challenge. Data are pooled from 2 independent experiments. Log-rank Mantel-Cox test was used to compare the PBS group with HKSTm-vaccinated and 30-week TAS2010-vaccinated groups, respectively.

The LAV strain TAS2010 carries attenuating mutations in the Embden-Meyerhof-Parnas (Δ*pfkA*Δ*pfkB*) and Entner-Doudoroff (Δ*edd*) pathways, but is still capable of replication and delivery of stimulatory signals and antigens in the host (S1A and S1B Fig). To begin separating the contribution of mucosal versus systemic immunity to TAS2010-induced vaccine protection, we first characterised the *in vivo* distribution and growth kinetics of TAS2010 in reference to the benchmark *S*. Typhimurium LAV, BRD509 (Δ*aroA*). Following oral gavage, TAS2010 persisted in the gut lumen for several weeks and was detected in the faeces, although faecal shedding of TAS2010 was markedly reduced compared with BRD509 once the bolus of the inoculum passed through the gut in the first few days (Fig 1A). One explanation for this growth difference in the gut lumen may relate to TAS2010’s slower growth rate under anaerobic conditions (S1C–S1E Fig). The other explanation is that, by lacking both Embden-Meyerhof-Parnas and Entner-Doudoroff pathways, the lower growth of TAS2010 in the gut might reflect their inability to catabolise gluconate in the GI tract.

We have previously observed that orally delivered TAS2010 invade the Peyer’s Patches and mesenteric lymph nodes (mLNs), then disseminate systemically to the spleen and liver within 24 hours [30]. TAS2010 grew to higher numbers in the spleen and liver than BRD509, although both LAVs were cleared or very nearly cleared over a similar time frame, i.e. within 10 weeks post-vaccination (Fig 1A). Subsequently, TAS2010-vaccinated mice were significantly better protected against challenge with wild-type *S*. Typhimurium SL1344 than those vaccinated with BRD509 (Fig 1B). TAS2010-vaccinated mice were able to better control SL1344 growth in systemic sites including the spleen and liver, whereas SL1344 levels in the mLNs and in faeces were similarly low in the two vaccinated groups, and in contrast to high burden in unvaccinated mice (Fig 1C). This result suggests that the potency of TAS2010-induced protection depends on systemic immunity rather than mucosal immunity.

To directly demonstrate the potency of systemic immunity independent of dissemination efficiency and local immune responses in the gut, we aimed to bypass the GI tract and delivered the LAV strains directly to the circulation via intravenous (i.v.) injection. Here, i.v. injected TAS2010 showed stronger growth in the spleen and liver than BRD509, particularly during the first 2–3 weeks, but the two LAV strains were also cleared to near, or below, detection levels by around week 10 post-vaccination (Fig 1D). Similar to the oral vaccination model, mice i.v. vaccinated with TAS2010 were significantly better protected against lethal challenge than those vaccinated with BRD509 (Fig 1E). Mice were still protected when challenged at least 30 weeks after vaccination with TAS2010 (Fig 1F), suggesting that TAS2010-induced immunity remains effective long term, and was more protective than vaccination with heat-killed *S*. Typhimurium (HKSTm) SL1344 (Fig 1F). Since systemic dissemination and replication of the LAV strains is a shared feature following orally or i.v. delivered inoculum, these data suggest that immunity developed at systemic sites is both capable and sufficient to confer protection against virulent *S*. Typhimurium introduced via the mucosal route. Hereafter, we have focused on using the i.v. infection model for investigating the mechanism of TAS2010-induced immunity at systemic sites.

### CD4^+^ T cells are required for effecting TAS2010-induced immunity against challenge

**Fig 2:**
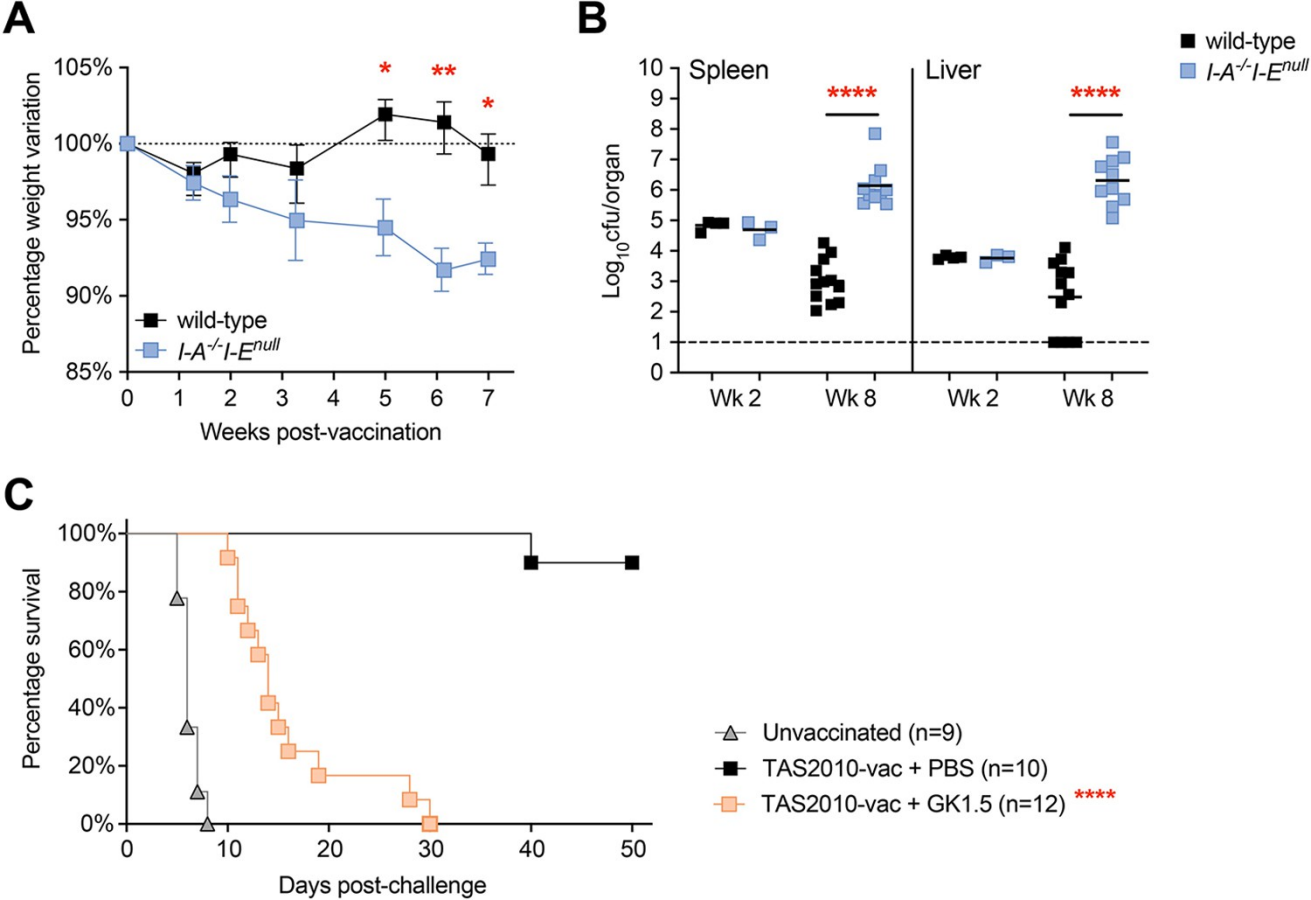
CD4^+^ T cell deficiency leads to impaired control of primary and secondary infection. Wild-type C57BL/6 or *I-A*^*-/-*^*I-E*^*null*^ mice were i.v. injected with 200cfu TAS2010. A) Infection-induced weight variation overtime is calculated as the percentage of initial body weight. Shown are mean ± SEM, n = 9–14, data pooled from 3 independent experiments. B) The bacterial load in the spleen and liver from individual mice is shown with geometric mean for each group at the indicated time points post-infection. C) C57BL/6 mice were intraperitoneally (i.p.) injected with αCD4 GK1.5 mAb or PBS at Wk 12 post-vaccination, a day prior to challenge with 10^7^cfu wild-type *S*. Typhimurium SL1344 by oral gavage. The depletion was maintained by i.p. injection with GK1.5 mAb twice weekly thereafter. Shown is the percentage of mice remaining protected at the indicated time points post-challenge. Data are pooled from 2 independent experiments. Log-rank Mantel-Cox test was used for statistical analysis between PBS-treated and CD4-depleted groups.

To assess the role of CD4^+^ T cells in TAS2010-induced immunity, we used two models of CD4^+^ T cell deficiency applied at the start of primary (vaccination) or secondary (challenge) infection, respectively. For assessing the requirement for CD4^+^ T cells during primary infection, MHC-II deficient mice (*I-A*^*-/-*^*I-E*^*null*^), which lack functional CD4^+^ T cells, were i.v. infected with TAS2010. These mice exhibited progressive weight loss (Fig 2A) and failed to control bacterial growth in the absence of CD4^+^ T cells (Fig 2B), succumbing to infection shortly after the week 8 time point. This indicates that CD4^+^ T cells are essential for the control and clearance of TAS2010 growth as the primary infection. For assessing the requirement for CD4^+^ T cells for immunity against challenge, neutralising mAb (clone GK1.5) was used to deplete CD4^+^ T cells in TAS2010-vaccinated wild-type mice immediately prior to challenge with SL1344, and immunity was abolished in the absence of CD4^+^ T cells (Fig 2C). These results demonstrate that CD4^+^ T cells are essential for controlling primary and secondary infections in this TAS2010 vaccination model.

### CD4^+^ T cells demonstrate robust activation and develop enhanced antigen-specific memory following vaccination with TAS2010

**Fig 3:**
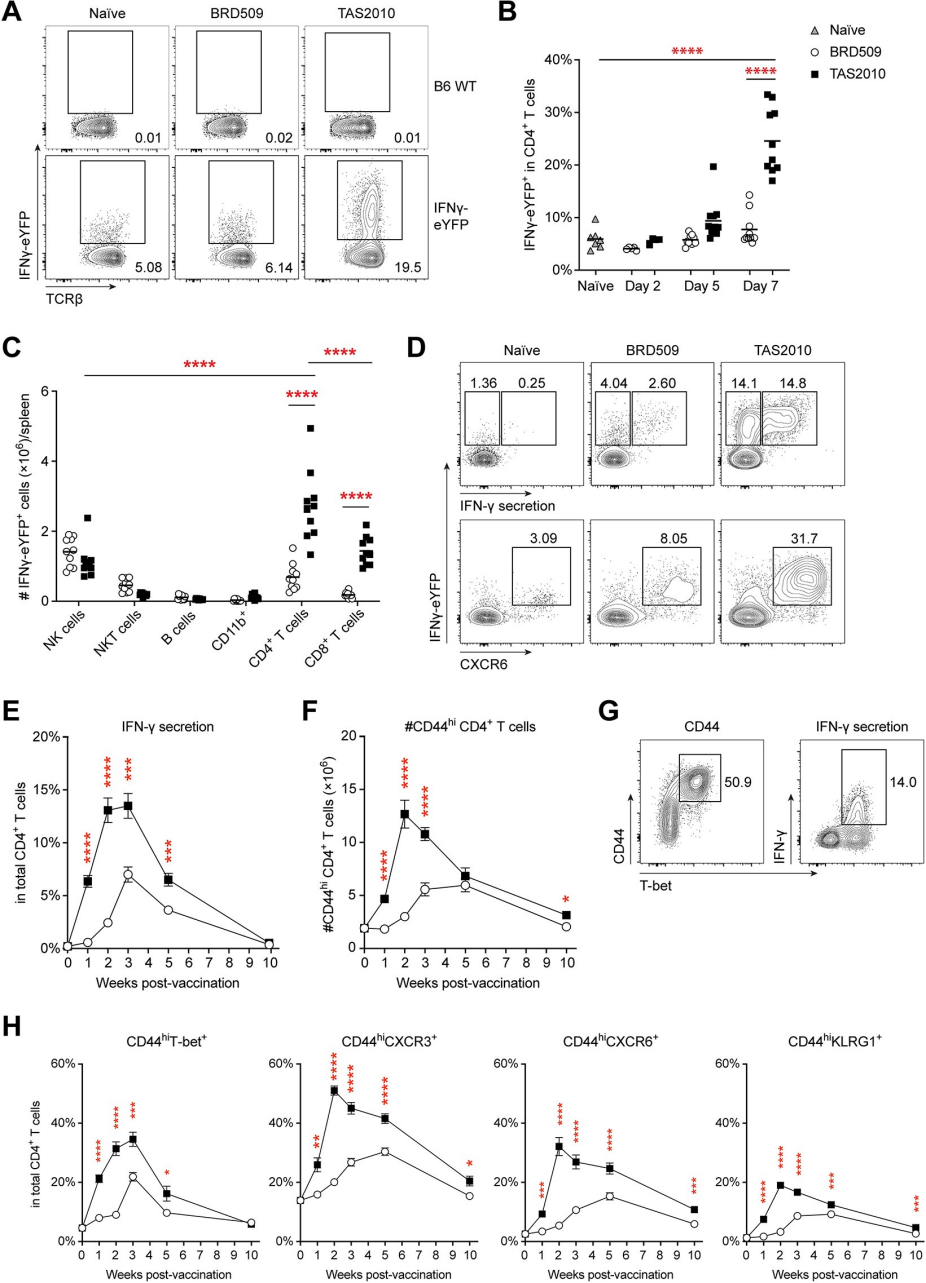
Vaccination with *S*. Typhimurium TAS2010 leads to robust Th1 activation in CD4^+^ T cells. Wild-type C57BL/6 or IFN-γ.eYFP reporter mice were either naïve or i.v. injected with 200cfu BRD509 or TAS2010, and the splenic CD4^+^ T cells were analysed at the indicated time points post-vaccination, where Wk 0 denotes data from naïve mice. **A, D, G)** Representative FACS plots show total viable CD4^+^ T cells in mice that were either naïve or at A) week 1 or D) week 2 post-vaccination with BRD509 or TAS2010; or G) week 2 post-vaccination with TAS2010. The frequency of gated cells is shown. **B, E, F, H)** The frequency of CD4^+^ T cells that B) expressed IFN-γ.eYFP reporter, E) secreted IFN-γ protein as determined by *ex vivo* secretion assay using diabodies, or H) stained for indicated activation/memory markers is shown. F) The number of CD44^hi^ CD4^+^ T cells was calculated per spleen. The mean or mean ± SEM is shown for each group at the indicated time points post-vaccination, and data are pooled from 2–4 independent experiments at each time point. Two-way ANOVA with Bonferroni’s post-tests were used for comparing the two vaccination groups at each time point. **C)** Spleen cells were identified as natural killer (NK) cells (CD3^-^NK1.1^+^), natural killer T (NKT) cells (CD3^+^NK1.1^+^), B cells (CD19^+^B220^+^), CD11b^+^ myeloid cells (CD3^-^CD19^-^CD11b^+^), CD4^+^ T cells (TCRβ^+^CD4^+^) or CD8^+^ T cells (TCRβ^+^CD8^+^) based on surface markers. The proportion of each cell subsets that expressed IFN-γ-eYFP was determined at day 7 post-vaccination. Shown are symbols for individual mice with group mean, data pooled from 2 independent experiments. Two-way ANOVA with Bonferroni’s post-tests were used twice: first for comparing the two vaccination groups for each immune cell subsets, then between cell subsets from TAS2010-vaccinated mice.

**Fig 4:**
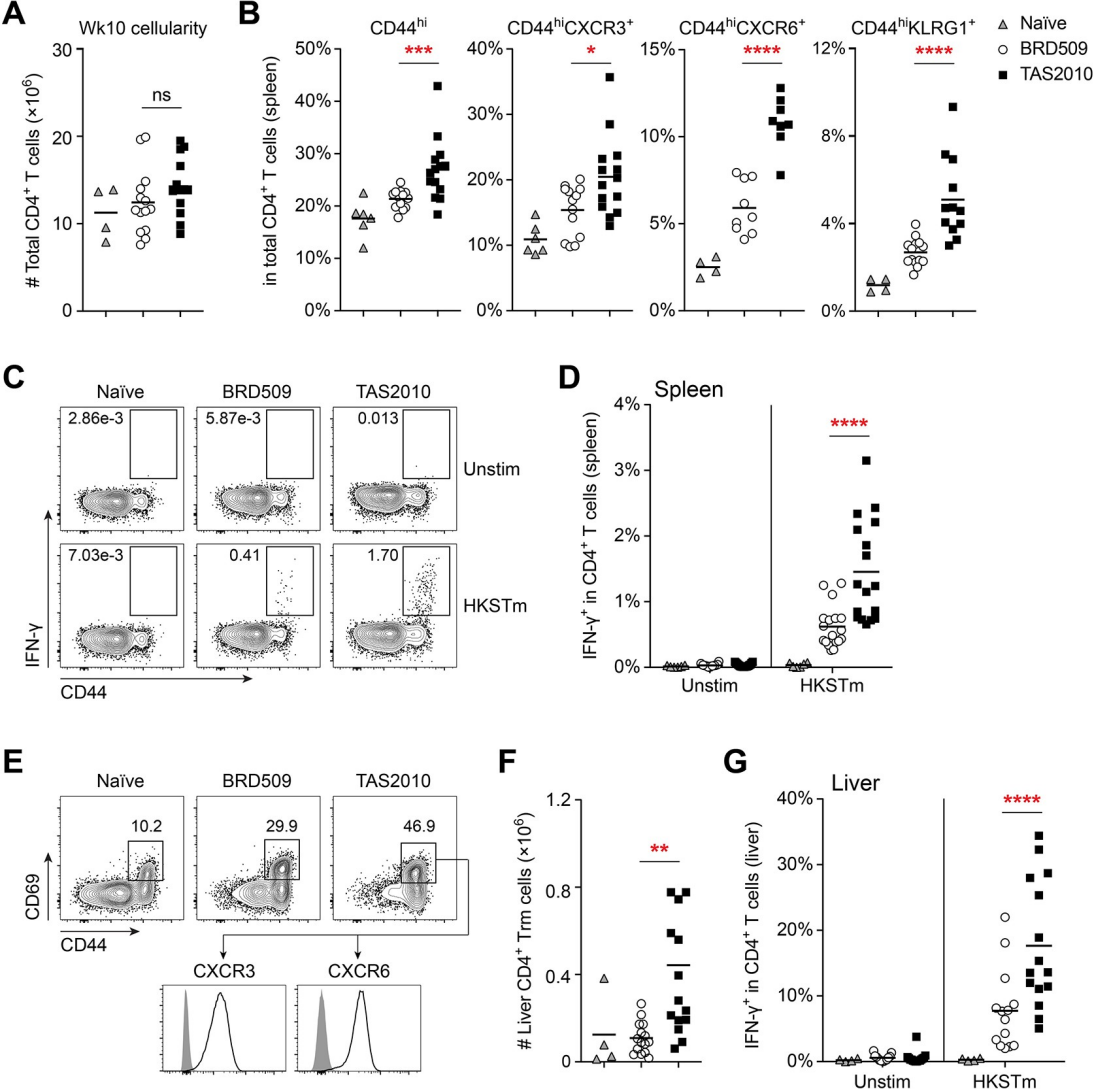
Vaccination with *S*. Typhimurium TAS2010 induces enhanced memory formation in CD4^+^ T cells. Wild-type C57BL/6 mice were either naïve or i.v. injected with 200cfu TAS2010 or BRD509. **A-D)** At week 10 post-vaccination, A) the total number of CD4^+^ T cells in the spleen, and B) the frequency of CD4^+^ T cells expressing indicated activation/memory markers are shown. C) Representative FACS plots and D) summarised data of the percentage of CD4^+^ T cells producing IFN-γ following *ex vivo* re-stimulation with 5×10^7^cfu heat-killed *S*. Typhimurium SL1344 (HKSTm), with unstimulated (unstim) cells set up as the control. Data from individual mice are shown as symbols with group mean, pooled from 3 independent experiments. Unpaired *t*-tests were used for statistical analysis between the two vaccination groups. **E-G)** Conventional CD4^+^ T cells were analysed at week 15 post-vaccination, with NKT cells excluded using a CD1d α-galactosylceramide (α-GalCer) tetramer. E) Representative FACS plots of conventional CD4^+^ T cells in the liver of naïve or vaccinated mice to show the frequency of CD44^hi^CD69^+^ CD4^+^ T cells; these cells (black line) also express high level of CXCR3 and CXCR6 compared to the unstained control (grey shade). F) The number of CD44^hi^CD69^+^ CD4^+^ T cells is calculated per liver. G) The frequency of liver-bound CD4^+^ T cells producing IFN-γ in response to *ex vivo* re-stimulation was determined. Data from individual mice are shown as symbols with group mean, pooled from 3 independent experiments. Unpaired *t*-tests were used for statistical analysis between the two vaccination groups.

To examine the development of CD4^+^ T cells from activation to the memory phase, we compared the kinetics of Th1 responses in mice vaccinated with TAS2010 or BRD509 over the 10-week vaccination time course. We used a reporter mouse that expressed eYFP under the transcriptional control of *Ifng* [31] for detecting early expression of IFN-γ as the signature cytokine for Th1 effector function. CD4^+^ T cells in TAS2010-vaccinated mice were activated rapidly (Fig 3A and 3B) and became the most abundant source of IFN-γ within 7 days (Fig 3C). IFN-γ transcription, as reported by eYFP, correlated with CXCR6 expression and largely overlapped with IFN-γ protein secretion, as detected using a diabody-based secretion/capture assay during a 1hr incubation *ex vivo* (Fig 3D). In TAS2010-vaccinated mice, the increased frequency of IFN-γ-production (Fig 3E) correlated with marked expansion of antigen-experienced CD44^hi^ CD4^+^ T cells (Fig 3F), the majority of which also expressed T-bet (Fig 3G), the master transcriptional factor for Th1 lineage differentiation [32]. The frequency of CD4^+^ T cells expressing CXCR3, CXCR6 and KLRG1 was also increased in TAS2010-vaccinated compared with BRD509-vaccinated mice, and this difference was most pronounced at week 2 (Fig 3H). In the memory phase (week 10 post-vaccination), mice of both vaccination groups harboured similar number of CD4^+^ T cells in the spleen (Fig 4A), but the frequency of CD4^+^ T cells expressing these activation markers was higher in TAS2010- compared with BRD509-vaccinated mice (Fig 4B). Upon *ex vivo* re-stimulation with HKSTm SL1344 as a whole-cell antigen mix, we observed a stronger, antigen-dependent recall response in CD4^+^ T cells from TAS2010-vaccinated compared with BRD509-vaccinated mice, as measured by IFN-γ production (Fig 4C and 4D). Taken together, these results indicate that a larger population of activated CD4^+^ T cells is retained in the spleen after vaccination with TAS2010, and these T cells most likely respond to *S*. Typhimurium-specific antigens.

We have previously described a CD4^+^ resident memory T (Trm) cell population in the liver uniquely capable of conferring protection against challenge with virulent *S*. Typhimurium [17]. These cells most likely originated from the spleen and were identified by constitutive expression of surface markers including CD44, CD69, CXCR3 and CXCR6, among others [17,22]. We found TAS2010-vaccinated mice also harboured an increased number of CD4^+^ Trm cells in the liver compared with BRD509-vaccinated mice after immunity developed (Fig 4E and 4F). Similar to CD4^+^ T cells in the spleen, liver-bound CD4^+^ T cells demonstrated antigen specificity as they readily produced IFN-γ upon *ex vivo* re-stimulation with HKSTm SL1344 (Fig 4G). Taken together, these results show that TAS2010 seeds robust, antigen-specific CD4^+^ T cell populations in systemic lymphoid and non-lymphoid tissues.

### Vaccination with *S*. Typhimurium LAVs triggers systemic inflammatory responses, resulting in maturation of inflammatory monocytes that promote Th1 responses

**Fig 5:**
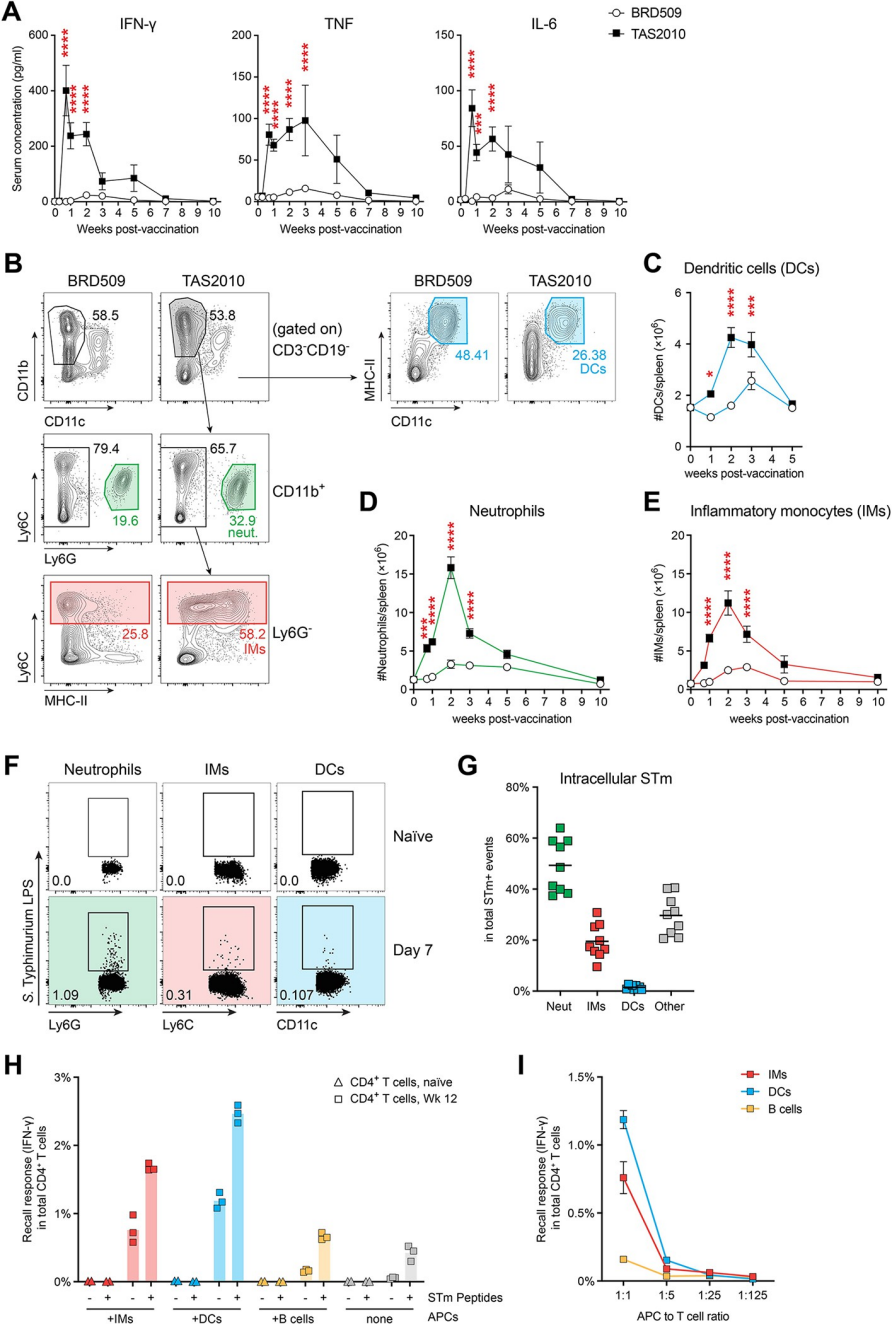
Inflammatory monocytes (IMs) are directly infected with *S*. Typhimurium and can present to antigen-specific CD4^+^ T cells. Wild-type C57BL/6 mice were either naïve or i.v. injected with 200cfu TAS2010 or BRD509, and the splenic CD4^+^ T cells were analysed at the indicated time points post-vaccination, where Wk 0 denotes data from naïve mice. **A)** The concentration of cytokines IFN-γ, TNF and IL-6 in the serum of wild-type C57BL/6 mice was determined using the cytometric bead array (CBA), shown as mean ± SEM, with data pooled from 2–4 independent experiments (n = 4–20). **B)** Representative FACS plots show gating strategies on CD11b^+^ myeloid cells. **C-E)** The numbers of C) CD11c^+^MHC-II^+^ conventional dendritic cells (DCs), D) CD11b^+^Ly6G^+^ neutrophils and E) CD11b^+^Ly6G^-^Ly6C^hi^ inflammatory monocytes (IMs) were calculated per spleen. Data are shown as group mean ± SEM for each time point, with data pooled from 3–6 independent experiments per time point (n = 9–29). Two-way ANOVA with Bonferroni’s post-tests were used for statistical analysis between the two vaccination groups. **F, G)** At day 7 post-vaccination with TAS2010, F) representative FACS plots show neutrophils, IMs and DCs all contained intracellular *S*. Typhimurium, and G) the distribution of intracellular *S*. Typhimurium (STm) among these cells was calculated as a percentage of total STm^+^ cells. Data are pooled from 2 independent experiments. **H, I)** CD4^+^ T cells were enriched from the spleens of either naïve or immune (Wk12 post-vaccination with TAS2010) mice and co-cultured with IMs, DCs or B cells sorted from the spleens of Wk1 TAS2010-infected mice. IFN-γ production by CD4+ T cells was measured using intracellular staining following a 18hr stimulation period. H) CD4^+^ T cells were re-stimulated by APC subsets at 1:1 ratio, with (+) or without (-) the addition of a 5-peptide mix (FliC_429-443_, GroEL_40-53_, LpdA_338-351_, SseI_268-280_ and SseJ_329-341_) that represent known CD4^+^ T cell epitopes in murine salmonellosis [20,51,55,82]. Three technical replicates and the mean are shown for each restimulation condition. I) CD4^+^ T cells were re-stimulated with decreasing APC to T cell ratio but without further addition of peptides, mean±SEM of three technical replicates are shown for each condition.

Since TAS2010 grew more vigorously *in vivo* than BRD509 (Fig 1D), we hypothesised that the abundance of *S*. Typhimurium-derived antigens and accessory signals (e.g. PAMPs) may be greater following TAS2010 vaccination, which may in turn augment T cell activation. In TAS2010-vaccinated mice, the peak of T cell activation coincided with strong inflammatory responses, including elevated circulating levels of IFN-γ, TNF and IL-6 (Fig 5A), and marked splenomegaly (S2 Fig). Substantial infiltration of dendritic cells (DCs) and CD11b^+^ myeloid cells, consisting predominantly of CD11b^+^Ly6G^+^ neutrophils and CD11b^+^Ly6G^neg^Ly6C^hi^ inflammatory monocytes (IMs), was also observed (Fig 5B–5E). Neutrophils and IMs represented the main cellular niche for *S*. Typhimurium based on intracellular staining for LPS (Fig 5F–5G). To address which of these MHC-II-expressing cells present *S*. Typhimurium antigens *in vivo*, we performed an *ex vivo* presentation assay by co-culturing equal numbers of FAC-sorted DCs, IMs or B cells from mice at 1 week after TAS2010 vaccination, with enriched CD4^+^ T cells from either naïve or immune (Wk 12) mice. CD4^+^ T cells from immune mice produced IFN-γ in an antigen- and dose-dependent manner upon stimulation by DCs and IMs but not B cells, and the addition of peptides for known CD4^+^ T cell epitopes further enhanced the recall response (Fig 5H–5I). These data demonstrate that IMs can access and present *S*. Typhimurium antigens. Since IMs were more numerous and more frequently infected than conventional DCs, it is likely that IMs carry important antigen presentation functions that amplify innate signals for T cell activation after they were primed by DCs.

**Fig 6:**
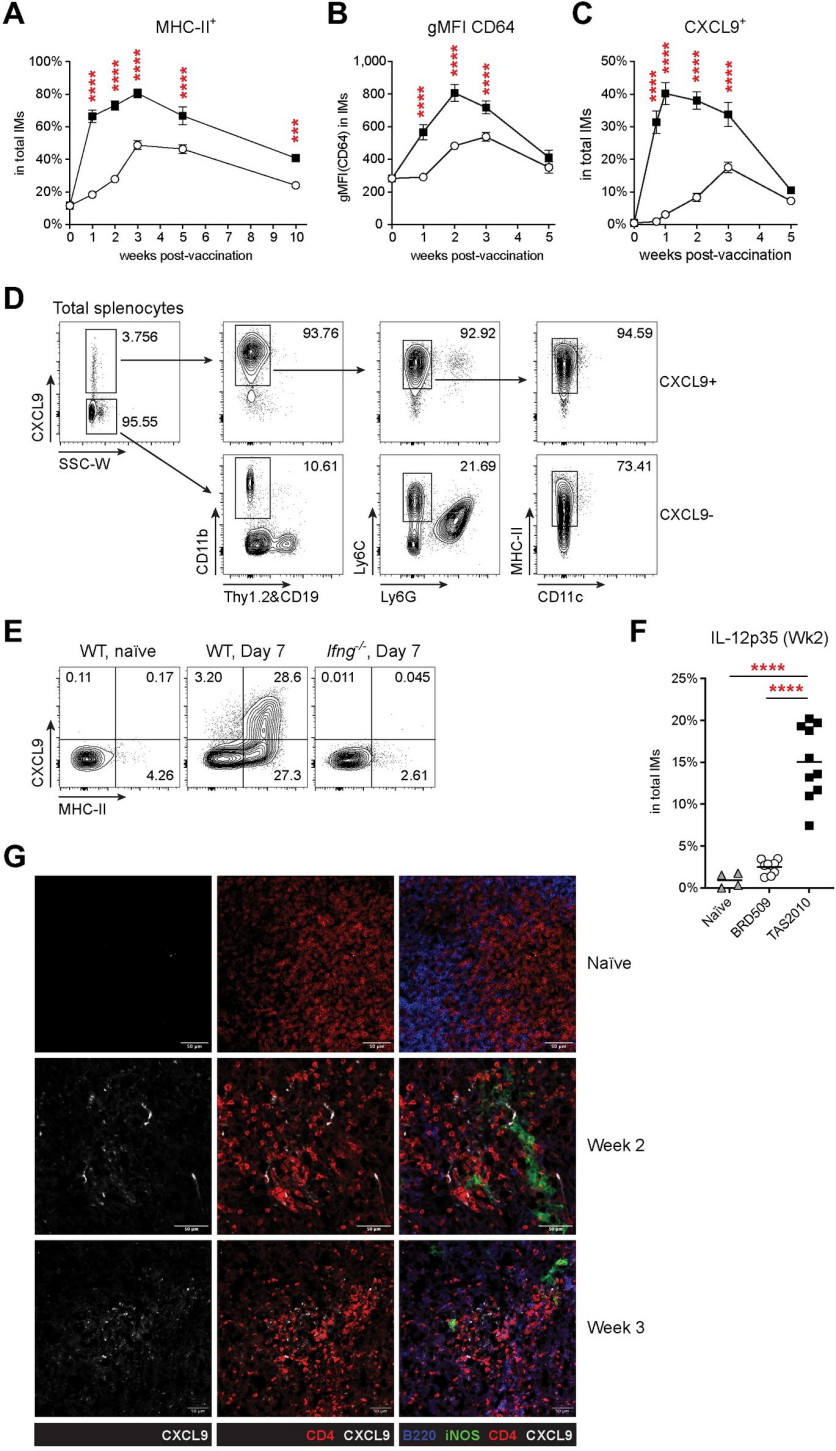
Inflammatory monocytes (IMs) show IFN-γ-dependent activation and become a potent source of CXCL9 and IL-12 following vaccination with *S*. Typhimurium TAS2010. Wild-type C57BL/6 mice were either naïve or i.v. injected with 200cfu TAS2010 or BRD509, and the splenic CD4^+^ T cells were analysed at the indicated time points post-vaccination, where Wk 0 denotes data from naïve mice. **A-C)** A) The frequency of MHC-II expression, B) geometric mean fluorescence intensity (gMFI) of CD64 expression, and C) the frequency of CXCL9 production was determined for IMs in the spleen. Data are shown as group mean ± SEM for each time point, with data pooled from 3–6 independent experiments per time point (n = 9–29). Two-way ANOVA with Bonferroni’s post-tests were used for statistical analysis between the two vaccination groups. **D)** Representative FACS plots showing staining of relevant markers in CXCL9-producing cells, using CXCL9-negative (CXCL9^-^) cells as the gating control. Plots show the vast majority of CXCL9-producing (CXCL9^+^) cells are MHC-II^+^ IMs. **E)** Representative FACS plots show IMs from mice that were either naïve or at day 7 post-vaccination with TAS2010. The expression of MHC-II and CXCL9 depends on IFN-γ. **F)** The percentage of IL-12p35-producing IMs was determined using intracellular staining for IL-12p35 after 4hr incubation with brefeldin A at 37°C *ex vivo*. Data are pooled from 2 independent experiments. One-way ANOVA with Bonferroni’s post-tests were used for statistical analyses. **G)** Representative spleen sections from C57BL/6 wild-type mice that were either naïve or vaccinated with TAS2010 for 2 or 3 weeks. Sections were stained with the indicated antibodies. White bars represent 50μm.

Next, the IM/Th1 nexus was further investigated. Compared with BRD509, vaccination with TAS2010 induced rapidly and substantially increased expression of MHC-II, CD64 and CXCL9 by IMs (Fig 6A–6C). CXCL9 is a ligand for CXCR3 expressed on Th1 cells, and plays an important role in Th1 recruitment [33]. Intracellular staining showed that the vast majority of CXCL9 in the spleen was produced by MHC-II^hi^ IMs (Fig 6D). The ability to interact with Th1 cells depends on IFN-γ signalling, as IMs from *Ifng*^-/-^ mice did not express MHC-II or CXCL9 (Fig 6E). We observed that IMs were able to produce IL-12 (Fig 6F), a pro-inflammatory cytokine that promotes potent Th1 differentiation by suppressing commitment to other Th lineages [34]. Confocal imaging of splenic sections from TAS2010-vaccinated mice showed co-localisation between CXCL9-expressing cells and CD4^+^ T cells (Fig 6G), demonstrating interaction between IMs and Th1 cells *in situ*. An elevated level of MHC-II expression was retained in the memory phase in TAS2010-vaccinated mice (Fig 6A), suggesting that IMs can provide an abundant and sustained source of antigens and stimulatory signals for maintaining activated CD4^+^ T cells into the memory phase. Together our data suggest that IMs play an important role in recruiting CD4^+^ T cells to the foci of infection via the CXCL9/CXCR3 axis and provide an important source of IL-12 for maintaining Th1 cells.

### Systemic inflammation and IM activation augment the development of Th1 responses and confer enhanced immunity against challenge

Our data so far led us to hypothesise that acute inflammation acts through infection-activated IMs to promote T cell activation and the development of Th1 memory. To test this hypothesis in a ‘loss-of-function’ model, we employed the *Ccr2*^*-/-*^ mice, which display deficient tissue recruitment of immature monocytes [35]. In line with previous reports [36,37], we observed that *Ccr2*^*-/-*^ mice were highly susceptible to TAS2010 (S3A Fig), suggesting a crucial role for monocytes in bacterial control. To our surprise, CD4^+^ T cells from *Ccr2*^*-/-*^ mice exhibited similar activation and Th1 differentiation compared with wild-type at day 7 post-infection (S3B Fig), and showed normal Th1 retention and recall response after TAS2010 was cleared with enrofloxacin treatment (S3C–S3F Fig). Further analysis showed that, although the total number of IMs was significantly reduced in TAS2010-infected *Ccr2*^*-/-*^ compared with wild-type mice (S4A Fig), the remaining *Ccr2*^*-/-*^ IMs showed rapid and robust activation (S4B Fig), presumably in response to a highly inflammatory environment characterised by markedly increased recruitment of neutrophils and elevated IFN-γ that is required for IM maturation (S4C and S4E Fig). In fact, TAS2010-infected *Ccr2*^*-/-*^ mice harboured significantly more IMs capable of stimulating T cells (i.e. MHC-II^+^ and CXCL9^+^) than BRD509-infected wild-type mice (S4F Fig), indicating that IMs in *S*. Typhimurium-infected *Ccr2*^*-/-*^ mice are reduced but not completely absent, as this has also been observed by others [37,38]. It is plausible that IMs may come from heterogeneous lineages and some may be recruited via CCR2-independent pathways, albeit less efficiently, and their activation is relative to the inflammatory signals. The residual IM population in *Ccr2*^*-/-*^ mice may still be sufficient for stimulating Th1 cells. However, since the depletion of IMs is incomplete in the *Ccr2* knockout, which also affected other monocyte subsets including DCs during *S*. Typhimurium infection (S4D Fig), we were unable to definitively address the role of IMs in Th1 development using this model alone.

**Fig 7:**
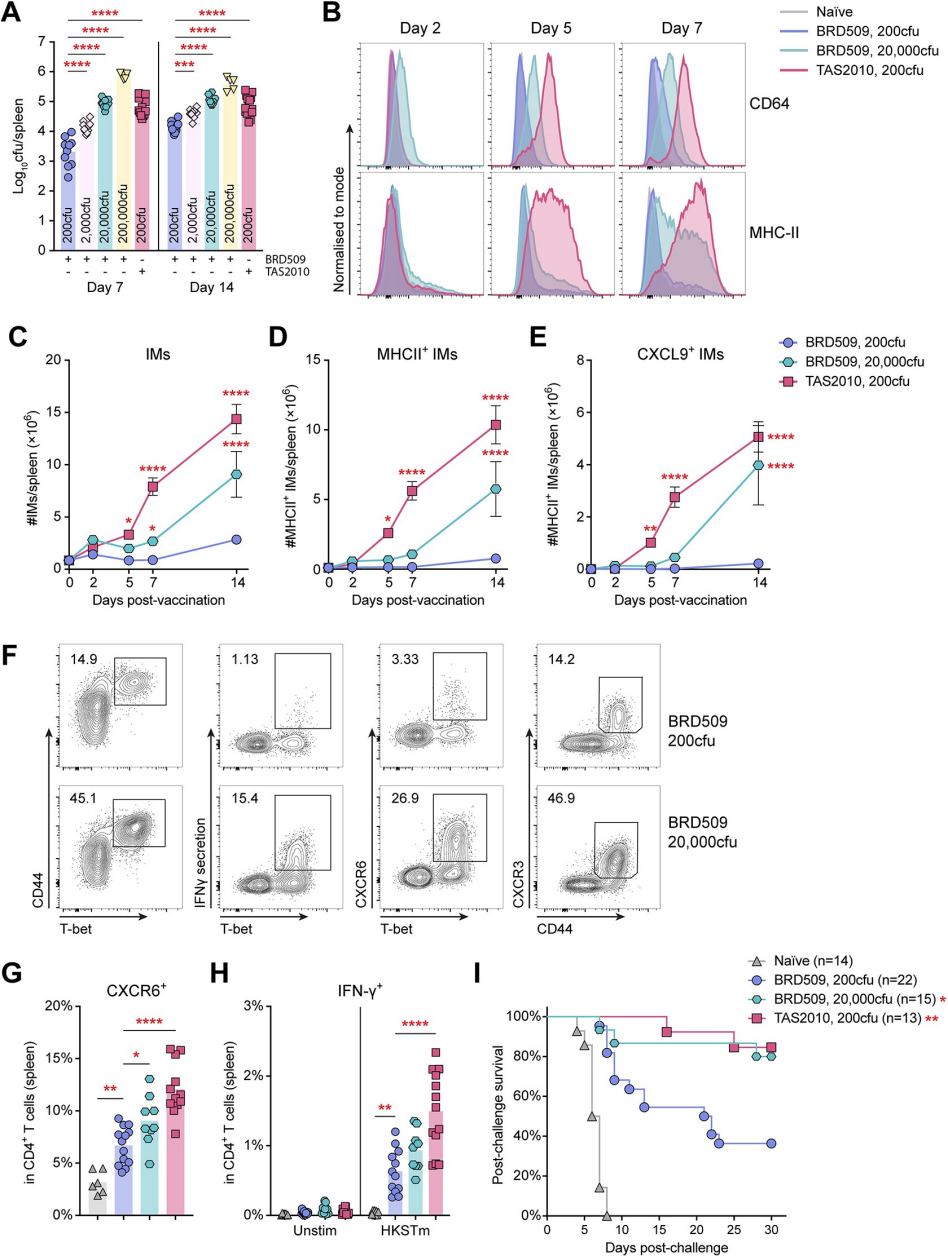
Escalated activation of IMs increased CD4^+^ T cell activation and led to improved BRD509-induced immune protection. Wild-type C57BL/6 mice were i.v. injected with 200cfu TAS2010 or escalating doses of BRD509, as indicated. **A)** The bacterial load in the spleen increased with the dose of BRD509 given. Data points are shown for individual mice with geometric mean for each group. Data are pooled from 3 independent experiments. One-way ANOVA with Bonferroni’s post-tests were used for statistical analyses between 200cfu BRD509 and other vaccination groups. **B-E)** B) Representative FACS histogram overlay show upregulation of CD64 and MHC-II expression in IMs at the indicated time points post-vaccination, in contrast to naïve mice. The numbers of C) total IMs, D) MHC-II^+^ IMs, and E) CXCL9^+^ IMs are calculated per spleen, and shown as mean ± SEM (n = 9–20). Data are pooled from 2–4 independent experiments. Two-way ANOVA with Bonferroni’s post-tests were used for statistical analysis between 200cfu BRD509 and other vaccination groups. **F)** Representative FACS plots show total viable CD4^+^ T cells in the spleen at week 2 post-vaccination. **G,H)** At week 10 post-vaccination, the frequency of CD4^+^ T cells G) expressing CXCR6, and H) producing IFN-γ in response to *ex vivo* re-stimulation was determined. Data from individual mice are shown as symbols with group mean, pooled from 3 independent experiments. One-way ANOVA with Bonferroni’s post-tests were used for statistical analysis between 200cfu BRD509 and other vaccination groups. **I)** At week 12 post-vaccination, mice were challenged with 10^7^cfu *S*. Typhimurium wild-type SL1344 by oral gavage. Shown is the percentage of mice remaining protected at the indicated time points post-challenge. Data are pooled from 2 independent experiments. Log-rank Mantel-Cox test was used for statistical analysis between 200cfu BRD09 and the other vaccination groups.

We then attempted to increase inflammation and IM activation in wild-type mice by inoculation with a larger vaccination dose of BRD509. We observed that tissue bacterial load increased with escalating doses of BRD509 delivered (Fig 7A), while the number of IMs and their expression of MHC-II, CD64 and CXCL9 also increased proportionally (Fig 7B–7E). Compared with mice receiving a low-dose (200cfu) of BRD509, mice vaccinated with a high-dose (20,000cfu) of BRD509 showed enhanced CD4^+^ T cell activation at week 2 post-vaccination (Fig 7F) and a trend for improved splenic retention of antigen-activated CD4^+^ T cells at week 10 post-vaccination (Fig 7G and 7H). Importantly, mice vaccinated with high-dose BRD509 were significantly better protected against challenge with virulent *S*. Typhimurium than those vaccinated with low-dose BRD509 (Fig 7I). These data strongly suggest that antigen availability associated with LAV growth is a key modulator of CD4^+^ T cell responses and the subsequent development of protective immunity. For the *S*. Typhimurium LAV models studied here, this can be optimally achieved either by using a larger dose of a slow-growing strain (e.g. high dose of BRD509) or a lower dose of a strain with a higher replication rate *in vivo* (e.g. TAS2010).

## Discussion

Despite their crucial role in orchestrating immunity against intracellular bacterial pathogens, relatively little is known about what is required for CD4^+^ T cells to form functional immunity and effect protection, a knowledge gap that significantly undermined the utility of these cells in vaccine development. The present study attempts to answer the question of what makes a LAV efficacious, focusing on CD4^+^ T cell immunity. Our data demonstrate that early inflammation can be beneficial for maximising the development of vaccine-induced CD4^+^ T cell immunity; mechanistically, CD11b^+^Ly6G^neg^Ly6C^hi^ IMs play an important role in providing spatial and functional cues that position CD4^+^ T cells to the foci of infection, from where CD4^+^ T cells can be sustained by having access to IL-12 and antigen stimulation. We found that the level of IM activation is proportional to inflammatory signals and can be modulated in response to bacterial load *in vivo*; increased IM recruitment have a direct impact on the quality and quantity of activated CD4^+^ T cells that are maintained for conferring protective immunity. Together our study highlights a beneficial role for inflammation in the development of CD4^+^ T cell-mediated immunity and identifies IMs as a potential target for enhancing T cell immunity to intracellular bacterial infection.

Our study raised several points around CD4^+^ T cell function with respect to timing and their localisation. The first point is early access to antigens. We found that CD4^+^ T cells from TAS2010-vaccinated mice exhibited earlier and stronger activation and expressed Th1 signature markers, including T-bet, CXCR3, and IFN-γ (Fig 3); this head start over BRD509-vaccinated mice was maintained into the memory phase and can be recalled using HKSTm as whole-cell antigens (Fig 4). This effect of early priming is consistent with a previous report that a minimum of 2 weeks of *S*. Typhimurium LAV growth *in vivo* is required for the full development of protective immunity [39], and shows that rapid onset of CD4^+^ T cell activation to LAV is an important first step towards effective immunity. The second point is the potential shift in T cell functionality between tissues over time. At the week 2 time point, bulk splenic cells can transfer immunity to a naïve, lymphopenic recipient in a CD4^+^ T cell-dependent manner [22]. This transferred immunity is more protective from TAS2010-vaccinated compared with BRD509-vaccinated donors [22], suggesting CD4^+^ T cells carried antimicrobial functions very early on, even though the effect of their depletion on systemic bacterial load does not become apparent until later in infection ([16] and Fig 2). Also starting around this early time point, a subset of Th1 cells likely migrated to the liver to seed a resident population capable of protection as immunity matures around 10–12 weeks, at which point immunity can be adoptively transferred from liver lymphocytes but not from spleen-derived cells [17,21]. One possible explanation is that splenic and liver CD4^+^ T cells diversify into functionally distinct subsets; and another explanation, favoured by our data, is that at the memory time point the liver has accumulated a larger population of antigen-specific Th1 cells, either through trafficking (e.g. via the CXCL16/CXCR6 axis) or because the liver microenvironment is more permissive for maintaining memory CD4^+^ T cells through mechanisms such as local antigen presentation [40]. Indeed, we observed antigen-specific CD4^+^ T cells in both the spleen and liver at the memory time point, but these cells are enriched in the liver (Fig 4) and can be transferred to effect protection [17]. Finally, since vaccination via the i.v. route was sufficient for controlling bacterial growth in the gut of immune mice (Fig 1), our data reaffirm the importance of central immunity in mucosal defence against faecal-orally transmitted pathogens [41] and demonstrate that the i.v. route is an especially effective delivery route for inducing robust tissue-resident memory T cells, as has been shown recently for BCG [42].

We have used IFN-γ production (or eYFP reporter activity) as a marker for early Th1 activation and, once the LAV strain is cleared, also for an antigen-specific recall response. The question remains whether the production of IFN-γ directly contributes to CD4^+^ T cell-mediated antimicrobial control. We observed that CD4^+^ T cells overtook NK cells [29,43] and non-cognate memory CD8^+^ T cells [44] to become the most abundant source of IFN-γ within the first week post-vaccination (Fig 3C). This time frame is probably too short for the majority of antigen-specific CD4^+^ T cells to reach peak frequency in a typical *S*. Typhimurium LAV infection but is more consistent with rapid activation of pre-existing, non-cognate CD4^+^ T cells, which respond to LPS stimulation and contribute to early infection control [45,46], coinciding with a critical period during which IFN-γ is required for antimicrobial control [12–14,16]. LPS also stimulates myeloid cells to produce IL-12 [47], which drives antigen-specific CD4^+^ T cells into the Th1 lineage to become highly productive for IFN-γ, at the expense of almost complete suppression of T follicular helper (Tfh) and germinal centre development [34,48,49]. It is plausible that responses from antigen-specific CD4^+^ T cells would eventually dominate over those from non-cognate CD4^+^ T cells as LAV bacteria are cleared. Although IFN-γ production is characteristic of cognate T cell activation, *S*. Typhimurium-specific CD4^+^ T cells are likely capable of IFN-γ-independent mechanisms because bacterial clearance is impaired when CD4^+^ T cells, but not IFN-γ, is depleted during late stages of primary infection [16,50]. Identifying the full range of antigenic targets for *S*. Typhimurium-specific CD4^+^ T cells would go a long way addressing this apparent dichotomy; although several CD4^+^ T cell epitopes have been identified [20,51–55], the responses are thought to be highly polyclonal and the majority of *S*. Typhimurium antigens recognised by CD4^+^ T cells remain undefined.

Fundamentally, *S*. Typhimurium LAVs occupy an intracellular growth niche and mimic important aspects of virulent infection [28]. These characteristics have a direct impact on which and how much antigens, in terms of T cell epitopes as well as endogenous PAMPs (e.g. LPS and flagellin), that LAVs can deliver to the host immune system, and could explain why a single dose of LAV is more effective in generating protective immunity than repeated administration of whole-cell, heat-killed bacteria (Fig 1F), consistent with previous reports [56,57]. In our study, both BRD509 and TAS2010 showed growth in systemic tissues for several weeks before clearance, but TAS2010 grew more vigorously, particularly when given to *Ifng*^*-/-*^ mice that are severely impaired for antimicrobial control (S5 Fig), thus supporting the view that manipulation of LAV virulence can lead to different vaccination outcomes. We increased the vaccination dose of BRD509 and found corresponding increases in tissue bacterial load and CD4^+^ T cell activation, which translated to improved immunity against challenge (Fig 7), strongly suggesting that the quantity of antigen delivery directly influences the level of T cell activation. At the cellular level, dose-dependent innate stimulations most likely translate to Th1 activation through CD11b^+^Ly6G^neg^Ly6C^hi^ IMs, the functional maturation of which required IFN-γ signalling (Fig 6E). Recent studies have shown that Ly6C^hi^ monocytes surround *S*. Typhimurium-confining granulomas in the spleen and recruit CD4^+^ T cells to the foci of infection by producing CXCL9 and CXCL10, both are ligands for CXCR3 expressed by Th1 cells [58]. Granulomas are absent in *Ifng*^*-/-*^ mice and, although granulomas are present in Th1 deficient (Tbet knockout) mice, they fail to contain *S*. Typhimurium within the granuloma [59]. We have observed that presentation-capable (i.e. MHC-II^+^) IMs were also the main producer of CXCL9 in the spleen (Fig 6D); these cells were directly infected and co-localised with CD4^+^ T cells *in situ* (Fig 6G), suggesting that IMs represent the convergent point between infection, antimicrobial control and T cell recruitment. When taken *ex vivo*, IMs were able to re-activate Th1 cells from immune mice without requiring additional antigens (Fig 5H and 5I), confirming that IMs have both the access and capacity to present *S*. Typhimurium antigens to Th1 cells. The spatial proximity between IMs and Th1 cells therefore creates an antigen-dependent feedback relay: while IFN-γ-activated IMs provide a source of antigen to promote Th1 differentiation and memory development, activated Th1 cells release IFN-γ to support IM maturation as well as contributing to antimicrobial control.

In addition to antigen dose, our data show that antigen persistence has a critical role in modulating the overall effectiveness of CD4^+^ T cell-mediated immunity. Whether a self-sustainable CD4^+^ T cell memory population exists *in vivo* is still debated because memory CD4^+^ T cells are thought to be poorly competitive with other lymphocyte subsets due to lower expression of receptors for ‘survival factors’ such as IL-7 and IL-15 [60]. Previous studies provided anecdotal evidence that the longevity of *S*. Typhimurium-specific CD4^+^ T cells may depend on how well their cognate antigens can persist *in vivo*: for instance, CD4^+^ T cells recognising flagellin, which is rapidly downregulated within hours of infection, showed premature contraction prior to bacterial clearance and formed poor memory [20,61,62]; whereas CD4^+^ T cells recognising SseJ, a *Salmonella* Pathogenicity Island-2 (SPI-2) effector protein with sustained expression, were maintained at a stable level longer term [20,63]. Mechanistically, it is proposed that a very low-level but ongoing antigen stimulation is required for maintaining a mixed population consisting of newly differentiated effectors and self-renewing memory CD4^+^ T cells in *S*. Typhimurium convalescent animals over months [64]. In this study, we repeatedly observed that mice were at the brink of bacterial clearance by week 10 post-vaccination with either TAS2010 or BRD509; while a few bacteria can still be detected in some but not all animals, no mice had circulating cytokines that would indicate ongoing immune activation at a systemic or pathological level (Fig 5A). However, we have noted that IMs continued to express a higher-than-background level of MHC-II at week 10 (Fig 6A), particularly in TAS2010-vaccinated mice, and this coincided with a higher frequency of antigen-experienced CD4^+^ T cells in the spleen expressing KLRG1 (Fig 4B), a marker typically associated with terminally differentiated effector T cells and antagonistic to memory development in CD8^+^ T cells [65], though its function in non-regulatory CD4^+^ T cells is less clear [66]. These data, combined with the observation that TAS2010-induced immunity can last for at least 30 weeks (Fig 1F), seemed to favour the interpretation that a low-level of antigen presentation is sustained in TAS2010-vaccinated mice, possibly by IMs, and contribute to improved vaccine protection.

The link between antigen dose, inflammation and immune protection significantly complicates real-world vaccine development, where the pursuit of efficacy needs to be very carefully balanced with the risk of overstimulation that can lead to adverse inflammatory reactions. Whether strong inflammatory/reactogenic responses is a friend or foe to vaccine efficacy has long been debated, the contentious point is not *whether*, but *what* and *how much*, inflammation is required for optimal induction of protective immunity. The replacement of whole-cell pertussis vaccines with the less reactogenic, acellular vaccines in high-income countries at the end of the 20^th^ century resulted in region-specific surges of pertussis cases in subsequent years. The waning immunity afforded by acellular pertussis vaccines was mostly attributed to a Th1 to Th2/17 immune profile shift and antigenic variation in the bacteria [67]. Indeed, increased inflammatory responses, particularly the Th1 type, are closely associated with the efficacy of vaccines against tuberculosis, Infleunza A, human papillomavirus and SARS-CoV-2, among others [42,68–70]. Conversely, immunosuppressive treatment for chronic inflammatory conditions can reduce the effectiveness of some vaccines [71,72]. In the case of *S*. *enterica*, balancing safety and efficacy has been a central issue in the clinical trials of several LAVs. *S*. Typhi LAV CVD908 (Δ*aroC*Δ*aroD*) was well tolerated but caused an asymptomatic vaccine bacteraemia, and attempts to further attenuate CVD908 by introducing additional mutations (e.g. *htrA*) minimised vaccine bacteraemia but also reduced immunogenicity [73]. Orally administered *S*. Typhimurium LAV WT05 (Δ*aroC*Δ*ssaV*) was immunogenic but caused prolonged faecal shedding [74]. In our murine model, we observed acute splenomegaly (S2 Fig) as well as significantly elevated IFN-γ, TNF, and IL-6 in the circulation of TAS2010-vaccinated mice (Fig 5A). Such a level of inflammation would be unsafe for direct use in humans despite the LAV’s efficacy; moving away from LAVs into purer and non-replicative vaccine formats might seem the logical next step but finding the adjuvant(s) that is both safe and effective remains a key challenge [75]. A recent study suggested that combined use of LPS and flagellin as adjuvants with a CD4^+^ T cell epitope gave good protection against virulent *S*. Typhimurium in mice [76], although the adjuvant dose required also triggered considerable morbidity.

Infectious diseases caused by intracellular bacterial pathogens continue to be a major cause of global morbidity and mortality. CD4^+^ T cells are essential for immunity against these infections and represent an important but under-exploited target in vaccine development, largely because the diversity of functions they can assume during an infection means that reproducing the array of signals for optimal development of the key lineages and in the appropriate tissue(s) in a vaccine setting is extremely challenging. Our study provides a critical analysis of LAV-induced CD4^+^ T cell immunity using a model of murine salmonellosis, and presents acute inflammation as a beneficial response to the development and maintenance of T cell immunity. Our study also highlights IMs as a convergent point for bridging innate inflammatory signals and the induction of adaptive immunity against intracellular bacterial infection. Further understanding of the molecular networks that underlie inflammation and antigen presentation functions in these cells may offer a path to eventually delineate adverse inflammatory responses from an efficacious vaccine.

## Materials and methods

### Ethics statement

All animal research conducted in this study was approved by the Animal Ethics Committee at the University of Melbourne, under project numbers 1413141, 1613898 and 2015171. All experiments were conducted in accordance with the National Health and Medical Research Council (NHMRC) Australian code for the care and use of animals for scientific purposes (2013).

### Bacterial strains and growth conditions

Live-attenuated *Salmonella enterica* serovar Typhimurium (*S*. Typhimurium) strains BRD509 (Δ*aroA*) [10] and TAS2010 (Δ*pfkA*Δ*pfkB*Δ*edd*)[29] were constructed on the SL1344 (wild-type)[77] genetic background. All *S*. Typhimurium strains were cultured in Luria-Bertani (LB) broth supplemented with 50μg/ml streptomycin, shaking at 180rpm and at 37°C overnight. For infection by oral gavage, the overnight culture was sub-cultured 1:1,000 in fresh LB broth and grown statically at 37°C overnight, washed twice in sterile PBS and then diluted to desired concentration. For intravenous (i.v.) infection, the overnight culture was sub-cultured 1:100 in fresh LB broth and grown shaking at 180rpm and at 37°C for 3hr to reach the mid-log phase (OD_600_ = 0.6–0.8), then stored frozen at -80°C in 10% glycerol. Aliquots were thawed immediately before use, washed twice in sterile PBS and diluted to desired concentration. For all infection experiments, the inoculum was plated on LB agar plates to confirm purity and the infection dose.

### Mouse infection

Age- and sex-matched C57BL/6 (B6) wild-type, *Ifng*-eYFP^in/in^ [31], *I-A*^-/-^*I-E*^*null*^ [78], *Ccr2*^*-/-*^ [35] and *Ifng*^-/-^ [79] mice were bred and housed at Biological Research Facility (BRF) at the Peter Doherty Institute for Infection and Immunity. For infection by oral gavage, mice were given 100μl 10% (w/v) sodium bicarbonate using a gavage needle immediately before the bacterial inoculum was delivered in 100μl PBS. For i.v. infection, the inoculum was injected into the lateral tail vein in 200μl PBS. For challenge experiments, mice that developed significant clinical symptoms, including sustained loss of >15% of initial body weight, persistent signs of distress and reduced wellbeing, were considered unprotected against virulent infection and euthanased as stipulated by our ethics guidelines, with the bacterial load in the spleen and liver assessed in order to confirm that morbidity was caused by infection. Infected tissues were homogenised using the Stomacher 80 Biomaster paddle blender (Seward), and serial dilutions were plated on LB agar plates with streptomycin (50μg/ml) to determine the bacterial load. Faecal pellets were collected, weighed, dissolved at 100mg/ml in PBS by vortexing at high speed for 10min and plated on XLD agar plates with streptomycin (50μg/ml).

### Depletion of CD4^+^ T cells *in vivo*

Mice were initially intraperitoneally (i.p.) injected with 250μg monoclonal antibody (mAb) GK1.5 (purchased from Bio X Cell) in 200μl PBS for the depletion of CD4^+^ T cells. Subsequently, the depletion was maintained by i.p. injection of 200μg GK1.5 mAb twice weekly.

### Spleen cell preparation

Single-cell suspension from the spleen was prepared either by pushing the spleen through a 70μm cell strainer, or by enzymatic digest in RPMI supplemented with 2% heat-inactivated foetal calf serum (FCS), 1mg/ml Type 3 collagenase (Worthington) and 0.14mg/ml DNase I (Roche) for 30min with agitation to preserve myeloid cells, which are more fragile and prone to cell death by mechanical dissociation. EDTA was added at a final concentration of 10mM in the final 10min of digest. At the end of dissociation or digest, aggregates were removed by passing through a 100μm nylon filter, then the cell suspension was underlaid with 1ml FCS and centrifuged at 400×*g* for 7min. Red blood cells were lysed in TAC buffer containing 17mM Tris and 140mM ammonium chloride at pH7.2. Cells were washed twice in FACS buffer (PBS with 2% FCS) containing 5mM EDTA before use in *ex vivo* assays or for staining with antibodies.

### Liver cell preparation

For protecting liver T resident memory (Trm) cells from apoptosis during preparation, mice were i.v. injected with 50μg anti-ARTC2 nanobody (Biolegend) in 200μl PBS 15min prior to euthanasia. Circulating cells were minimised by perfusing the mouse with 10ml sterile PBS through the hepatic portal vein following euthanasia and processed as previously described [80]. Briefly, the liver was pushed through a 70μm cell strainer, washed twice in FACS buffer, then leukocytes were separated from hepatocytes by centrifugation in 33% isotonic Percoll at 700×*g* and at RT for 12min. TAC buffer was used to lyse red blood cells and remaining leukocytes were washed twice in FACS buffer containing 5mM EDTA before use in *ex vivo* assays or for staining with antibodies.

### Flow cytometry

Aliquots of single-cell suspension were incubated with Fc block (BD Bioscience) for 15min at 4°C in FACS buffer containing 5mM EDTA, and then stained with conjugated mAbs against surface markers and with fixable viability dye e780 (eBioscience) for excluding dead cells. For intracellular staining, cells were fixed and permeabilised using the FoxP3 staining kit (eBioscience) according to manufacturer’s instructions. The mAbs against the following surface and intracellular markers were purchased from BD Bioscience, eBioscience or Biolegend: CD3 (17A2), CD4 (GK1.5 or RM4-5), CD8 (53–6.7), CD11b (M1/70), CD11c (HL3), CD19 (1D3), CD44 (1M7), CD45R/B220 (RA3-6B2), CD49b (DX5), CD62L (MEL-14), CD64 (X54-5/7.1), CD69 (H1.2F3), CXCL9 (MIG-2F5.5), CXCR3 (CXCR3-173), CXCR6 (SA051D1), FoxP3 (FJK-16s), IFN-γ (XMG1.2), IL-12p35 (4D10p35), Ly6C (AL-21), KLRG1 (2F1), Ly6G (1A8), MHC-II (M5/114.15.2), NK1.1 (PK136), T-bet (4B10), TCRβ (H57-597) and TNF (MP6-XT22). CD1d α-galactosylceramide (α-GalCer) tetramer was obtained from Dale Godfrey (The University of Melbourne). Calibration beads (BD Bioscience) were used for calculating cell numbers and samples were analysed using LSRII or LSRII Fortessa (BD Bioscience). Data were then analysed using the FlowJo software (TreeStar).

### Re-stimulation with heat-killed *S*. Typhimurium (HKSTm)

*S*. Typhimurium SL1344 was grown statically in LB broth overnight, washed once in PBS and heat-killed by incubation in a 60°C water bath for 1hr. An aliquot was removed before heat-kill to estimate bacterial number and an aliquot after was used to confirm non-viability. For *ex vivo* re-stimulation, approximately 5×10^7^cfu HKSTm were added to 1–2 million cells from the spleen or liver in round-bottom 96-well plates and in complete T cell media (RPMI containing 10% FCS, 2mM L-Glutamax, 10mM HEPES, 2mM sodium pyruvate, 1× MEM non-essential amino acids, 50μM 2-mercaptoethanol, 50U/ml penicillin and 50μg/ml streptomycin, all purchased from Gibco), incubated at 37°C and 5% CO_2_ for 14-16hr, then brefeldin A (GolgiPlug, BD Bioscience) was added for another 4hr for trapping IFN-γ inside of the cell.

### Detection of *ex vivo* IFN-γ secretion

CD4^+^ T cells spontaneously secrete IFN-γ during infection with *S*. Typhimurium and this was measured directly *ex vivo* using the mouse IFN-γ secretion assay–detection kit (Miltenyi). Briefly, cells were firstly incubated with a diabody binding to cell surface CD45 at 4°C for 15min, then cells were gently agitated in RPMI supplemented with 2% FCS at 37°C for 1hr such that secreted IFN-γ is captured by the diabody. Cells were then stained with a fluorescently-conjugated mAb against IFN-γ for detecting secreting cells.

### Intracellular staining for *S*. Typhimurium LPS

Spleen cells were prepared in FACS buffer containing 50μg/ml gentamicin for inhibiting extracellular *S*. Typhimurium from entering cells during preparation. Cells were stained for surface markers and then fixed and permeabilised using the FoxP3 staining kit (eBioscience) according to manufacturer’s instructions. Monoclonal *S*. Typhimurium LPS (clone 1E6, Thermo Fisher) was conjugated using the Alexa Fluor 647 Protein Labelling Kit (Thermo Fisher) and used for intracellular labelling of *S*. Typhimurium. We have verified that the antibody stained all three strains of *S*. Typhimurium used in this study, but does not cross-react with *E*. *coli*.

### *Ex vivo* presentation assay

Spleens from wild-type C57BL/6 mice that were either naïve or at Wk 12 post-vaccination with *S*. Typhimurium TAS2010 were pooled, made into a single-cell suspension and were negatively enriched for CD4^+^ T cells to 70–80% purity using a mAb cocktail containing clones Ter119, M5/114, M1/70 and F4/80. Enriched CD4^+^ T cells were plated at 5×10^5^ cells per well in round-bottom 96-well plates in complete T cell media. For presentation, APC subsets were sorted into inflammatory monocytes (CD11b^+^Ly6G^neg^Ly6C^hi^), dendritic cells (CD11c^+^MHC-II^+^) or B cells (CD19^+^B220^+^) by FACS Aria III (BD Bioscience) from Wk 1 TAS2010-infected mice. Enriched CD4^+^ T cells and sorted APCs were co-cultured at various APC to T cell ratio, and received either no further antigen stimulation *ex vivo* or a 5-peptide mix (FliC_429-443_, GroEL_40-53_, LpdA_338-351_, SseI_268-280_ and SseJ_329-341_) at 1μg each/well. The co-cultures were incubated at 37°C and 5% CO_2_ for 14hr before brefeldin A (GolgiPlug, BD Bioscience) was added for another 4hr for trapping IFN-γ inside of the cell.

### Cytometric bead array (CBA) for analysing serum cytokines

Blood was collected from mice immediately after euthanasia by CO_2_ asphyxiation, allowed to clot at RT for 3hr, and serum was separated by centrifugation. The mouse inflammation CBA kit (BD Bioscience) was used for multiplex detection of IL-6, IL-10, MCP-1, IFN-γ, TNF and IL-12p70 following manufacturer’s instructions. A minimum of 300 events was collected for each analyte on FACS CantoII (BD Bioscience), and data were analysed using the FCAP Array software, v3 (SoftFlow).

### Immunohistology

Spleen biopsies were submerged in PLP buffer (1% paraformaldehyde, 75mM sodium phosphate monobasic, 75mM disodium phosphate, 50mM L-Lysine and 10mM sodium periodate) overnight at 4°C, washed three times in PBS and then dehydrated in 30% (w/v) sucrose overnight. Fixed tissues were then stored at -80°C in OCT Compound (Tissue-Tek) until use. A Leica cryostat was used to cut fixed and frozen tissues at 10μm thickness and mounted onto SuperFrost Plus slides. Sections were permeabilised with 0.3% Triton X-100, 0.1M glycine, 0.1% cold fish skin gelatin and 1% BSA in PBS for 10 min, blocked with serum-free protein block (Agilent) for 1hr, and then stained with fluorescently-conjugated mAbs against iNOS Alexa Fluor 488 (CXNFT, ThermoFisher), CXCL9 eFluor 660 (MIG-2F5.5, ThermoFisher), CD4 CF-594 (RM4-5, BD Bioscience) and B220 Pacific Blue (RA3-6B2, BioLegend) for 1hr, all at room temperature. Stained tissue sections were mounted with ProLong Gold Antifade Mountant (ThermoFisher) and imaged using a Zeiss LSM780 confocal microscope. Images were analysed using the Fiji software [81].

### Statistical analyses

Graphpad Prism software, v9 (Dotmatics) was used for plotting the graphs and for conducting statistical analyses. Survival data between groups in challenge experiments were compared using the log-rank Mantel-Cox test. Unpaired *t*-tests were used to compare numerical data between the two vaccination groups, and adjusted for comparison over multiple time points using the Holm-Sidak’s method. One-way or two-way ANOVA with Bonferoni’s post-tests were used for comparing all groups with each other, or, where indicated, all groups with a designated group. *P*-values greater than 0.05 were considered not statistically significant (ns); statistical significance was noted as the following: * *p*<0.05, ** *p*<0.01, *** *p*<0.001, **** *p*<0.0001.

## Supporting information

S1 Fig *S*. Typhimurium strain TAS2010 (Δ*pfkA*Δ*pfkB*Δ*edd*) is attenuated for *in vivo* growth due to genetic disruptions in carbon metabolism pathways. **A)** Schematic diagram showing key metabolic steps in the Embden-Meyerhof-Parnas (EMP) and Entner-Doudoroff (ED) pathways, which are blocked in *S*. Typhimurium strain TAS2010 (Δ*pfkA*Δ*pfkB*Δ*edd*). Shaded boxes represent metabolites and arrows show the physiological direction of enzymatic reactions. Reactions blocked by the mutations are shown with a cross. DHAP, Dihydroxyacetone phosphate; Fru-6-P, D-Fructose 6-phosphate; Fru-1,6-P2, D-Fructose 2,6-bisphosphate; GADP, Glyceraldehyde 3-phosphate; Glu-6-P, D-Glucose 6- phosphate; Glul-6-P, D-Glucono-1,5-lactone 6-phosphate; Gln-6-P, D-Gluconate 6-phosphate; KDPG, 2-keto-3deoxy-6-phosphogluconate;PEP, Phosphoenolpyruvate; Ribu-5-P, D-Ribulose 5-phosphate; Rib-5-P, D-Ribose 5-phosphate. **B)** Wild-type C57BL/6 mice were given an oral gavage of 8×10^6^cfu of indicated strain of *S*. Typhimurium, and the bacterial load in the spleen and liver was analysed at day 6 post-infection (dotted line represents detection limit). Note here the bacterial load in the spleen and liver was lower than shown in Fig 1A because a lower infection dose was used. The geometric mean of each group is shown, data are pooled from 3 independent experiments. One-way ANOVA with Bonferroni’s post-tests were used for statistical analysis in each organ. **C-E)** *S*. Typhimurium BRD509 (○) or TAS2010 (◼) were grown to stationary phase in LB broth with streptomycin (50μg/ml) overnight and then normalised to OD_600_ of 0.8. The normalised culture was sub-cultured 1:100 into fresh LB broth and grown for 24hr at 37°C in 96-well plates, with absorbance at 600nm measured every hour by the CLARIOStar plate reader. The cultures were grown C) aerobically, with shaking at 300rpm in 200μl LB broth per well, D) anaerobically, by static growth in 200μl LB broth plus 80μl mineral oil overlay per well, or E) anaerobically, by static growth in 300μl LB broth which filled the well fully. The mean of six technical replicates is shown, data representative of 2 independent experiments.
(TIF)

S2 Fig Vaccination with *S*. Typhimurium leads to transient splenomegaly that is resolved as the LAV strain is cleared. Wild-type C57BL/6 mice were i.v. vaccinated with 200cfu TAS2010 (◼) or BRD509 (○). A) Representative spleens at week 2 post-vaccination. B) Spleen weight was measured at the indicated time points post-vaccination, mean ± SEM shown. Data are pooled from 2–4 independent experiments (n = 9–20), at the indicated time points post-vaccination, where Wk 0 denotes data from naïve mice. Two-way ANOVA with Bonferroni post-tests were used for statistical analysis.
(TIF)

S3 Fig *Ccr2*^*-/-*^ mice are more susceptible to infection with *S*. Typhimurium TAS2010, but exhibit normal CD4^+^ T cell responses. Wild-type C57BL/6 (black) or *Ccr2*^-/-^ (orange) mice were either naïve or i.v. injected with 200cfu TAS2010. **A, B)** A) The bacterial load in the spleen and liver (geometric mean shown) and B) the number of activated CD4^+^ T cell subsets in the spleen were quantified at day 7 post-infection. IFN-γ secretion was measured using a diabody-based, *ex vivo* IFN-γ secretion assay. **C-F)** Mice were treated with 0.2mg/ml enrofloxacin (ENR) in the drinking water from Wk 1 until analysis at Wk 5 post-infection, as shown in C) the schematic diagram. D) Treated mice cleared bacteria in the spleen (geometric mean shown). The number of splenic CD4^+^ T cells that E) stained positive for T-bet intracellularly or F) produced IFN-γ following 18 hr of *ex vivo* re-stimulation with HKSTm was quantified. Data from individual mice are shown as symbols with group mean, pooled from 2–3 independent experiments. One-way ANOVA with Bonferroni’s post-tests were used for statistical analyses.
(TIF)

S4 Fig IMs from *Ccr2*^*-/-*^ mice show potent activation in response to *S*. Typhimurium TAS2010. Wild-type C57BL/6 (black) or *Ccr2*^-/-^ (orange) mice were either naïve or i.v. injected with 200cfu TAS2010 or BRD509. Mice were analysed at day 7 post-infection. **A, C-D)** The number of A) CD11b^+^Ly6G^neg^Ly6C^hi^ inflammatory monocytes (IMs), C) CD11b^+^Ly6G^+^ neutrophils and D) CD11c^+^MHC-II^hi^ conventional DCs were quantified. **B, F)** IMs were stained for MHC-II (surface) and CXCL9 (intracellular) and analysed by flow cytometry. B) Representative staining profiles are shown. F) the number of MHC-II- or CXCL9-expressing IMs was quantified. **E)** Serum concentration of IFN-γ was determined using the cytometric bead array (CBA). Data from individual mice are shown as symbols with group mean, pooled from 3–4 independent experiments. Brown-Forsythe and Welch ANOVA tests with Dunnett T3 corrections (a variation of One-way ANOVA that does not assuming equal variance) were used for statistical analyses.
(TIF)

S5 Fig Mice with reduced immune control over *S*. Typhimurium growth are highly susceptible to infection with *S*. Typhimurium. Wild-type C57BL/6 (black) or *Ifng*^-/-^ (pink) mice were i.v. injected with 200cfu TAS2010 (square) or BRD509 (circle). A) The bacterial load in the spleen and liver from individual mice is shown with geometric mean for each group at day 7 post-infection. Data are pooled from 2 independent experiments. Two-way ANOVA with Bonferroni’s post-tests were used twice: first for comparing the two mouse genotypes, then for comparing the same genotype of mice infected with different *S*. Typhimurium strains. B) Shown is the percentage of mice remaining that were not considered moribund at the indicated time points post-infection. Data are pooled from 2 independent experiments. Log-rank Mantel-Cox test was used for statistical analysis.
(TIF)
