# Supplementary figures and images for: Vaccine-induced inflammation and inflammatory monocytes promote CD4+ T cell-dependent immunity against murine salmonellosis
Source: PLoS Pathog. 2023 Sep 21;19(9):e1011666. doi: 10.1371/journal.ppat.1011666 (PMC10547166; doi:10.1371/journal.ppat.1011666)

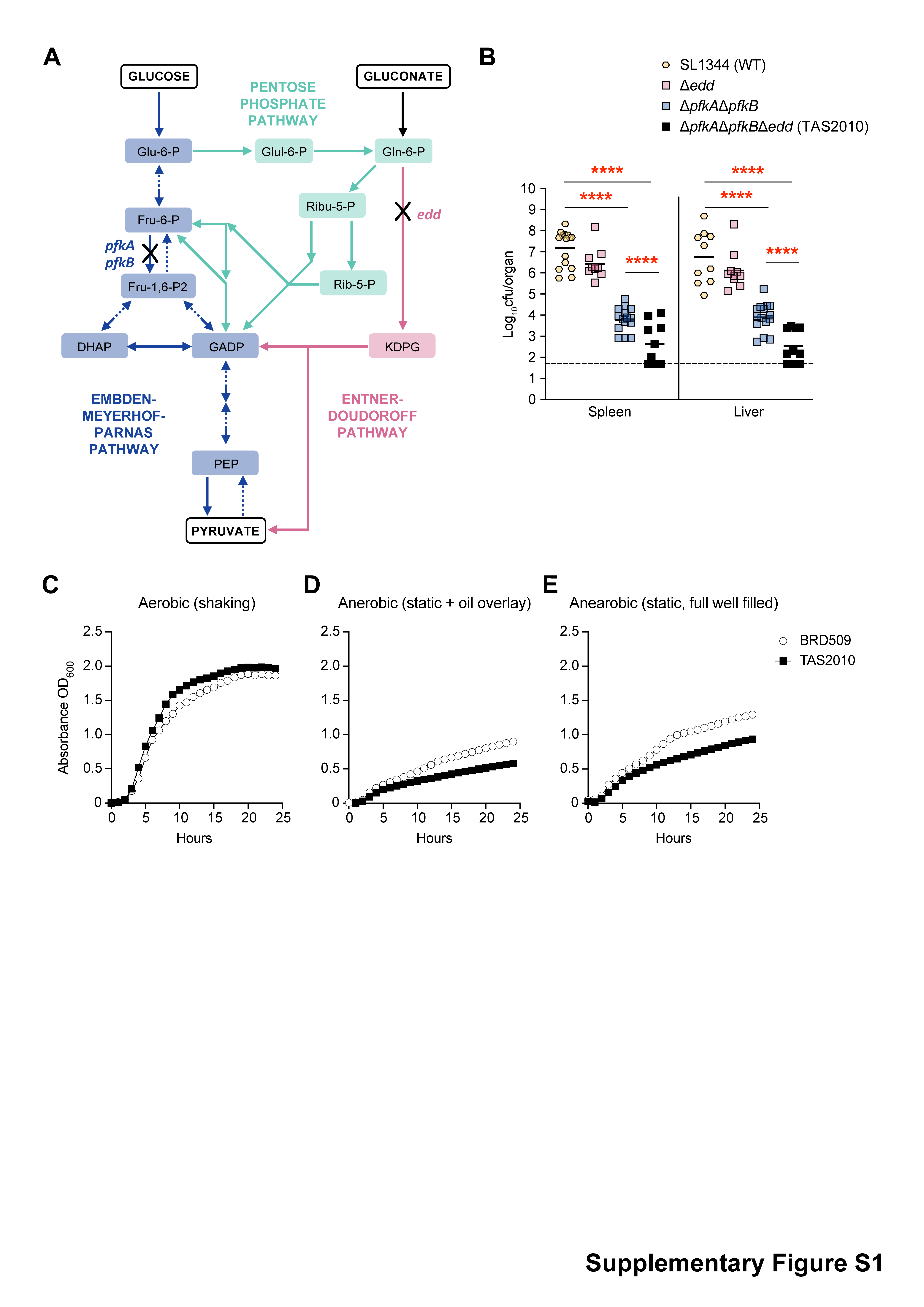

Supplement: S1 Fig — A) Schematic diagram showing key metabolic steps in the Embden-Meyerhof-Parnas (EMP) and Entner-Doudoroff (ED) pathways, which are blocked in S. Typhimurium strain TAS2010 (ΔpfkAΔpfkBΔedd). Shaded boxes represent metabolites and arrows show the physiological direction of enzymatic reactions. Reactions blocked by the mutations are shown with a cross. DHAP, Dihydroxyacetone phosphate; Fru-6-P, D-Fructose 6-phosphate; Fru-1,6-P2, D-Fructose 2,6-bisphosphate; GADP, Glyceraldehyde 3-phosphate; Glu-6-P, D-Glucose 6- phosphate; Glul-6-P, D-Glucono-1,5-lactone 6-phosphate; Gln-6-P, D-Gluconate 6-phosphate; KDPG, 2-keto-3deoxy-6-phosphogluconate;PEP, Phosphoenolpyruvate; Ribu-5-P, D-Ribulose 5-phosphate; Rib-5-P, D-Ribose 5-phosphate. B) Wild-type C57BL/6 mice were given an oral gavage of 8×10^6^cfu of indicated strain of S. Typhimurium, and the bacterial load in the spleen and liver was analysed at day 6 post-infection (dotted line represents detection limit). Note here the bacterial load in the spleen and liver was lower than shown in Fig 1A because a lower infection dose was used. The geometric mean of each group is shown, data are pooled from 3 independent experiments. One-way ANOVA with Bonferroni’s post-tests were used for statistical analysis in each organ. C-E) S. Typhimurium BRD509 (○) or TAS2010 (◼) were grown to stationary phase in LB broth with streptomycin (50μg/ml) overnight and then normalised to OD600 of 0.8. The normalised culture was sub-cultured 1:100 into fresh LB broth and grown for 24hr at 37°C in 96-well plates, with absorbance at 600nm measured every hour by the CLARIOStar plate reader. The cultures were grown C) aerobically, with shaking at 300rpm in 200μl LB broth per well, D) anaerobically, by static growth in 200μl LB broth plus 80μl mineral oil overlay per well, or E) anaerobically, by static growth in 300μl LB broth which filled the well fully. The mean of six technical replicates is shown, data representative of 2 independent experiments. (TIF) [file ppat.1011666.s001.tif]

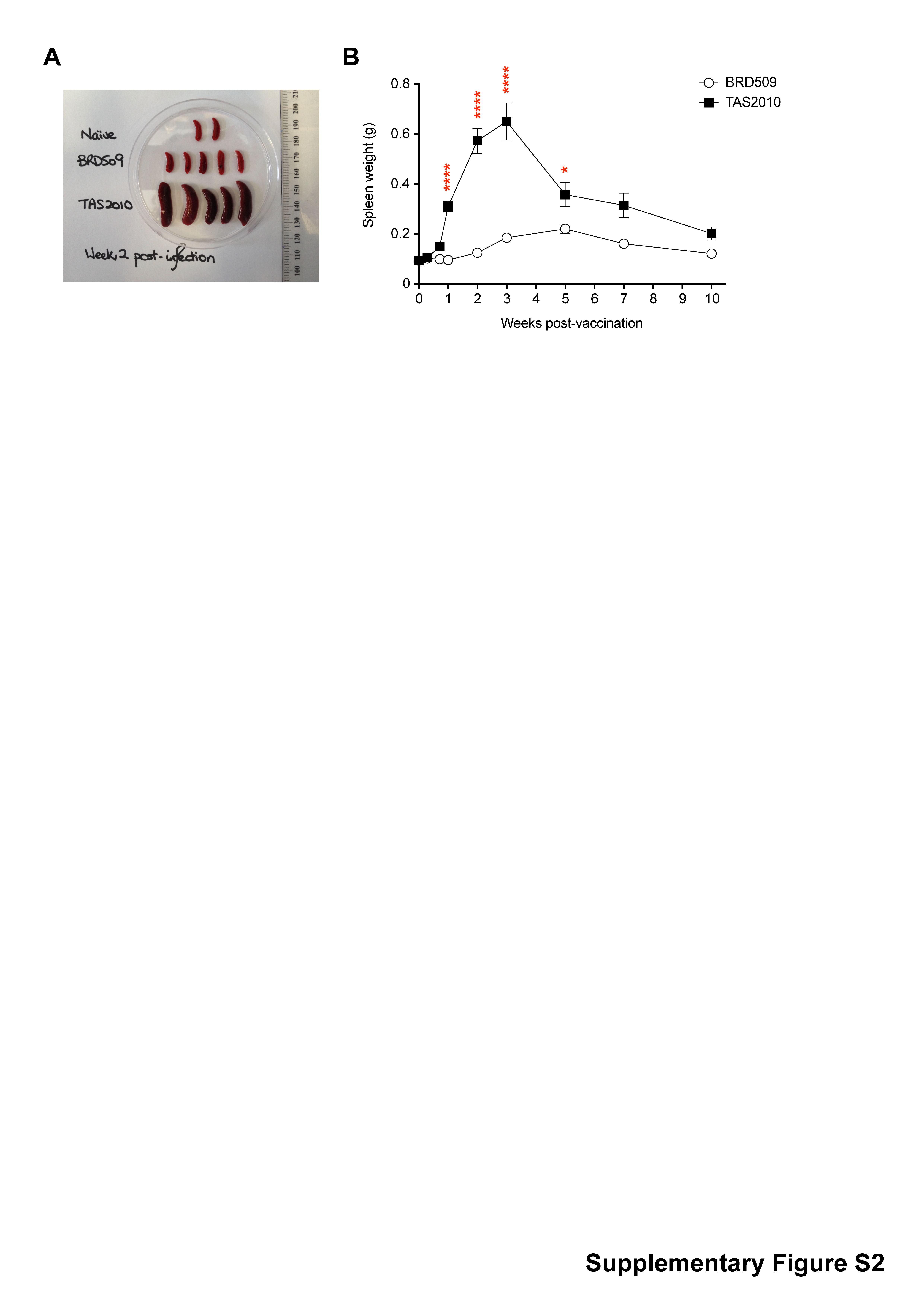

Supplement: S2 Fig — Wild-type C57BL/6 mice were i.v. vaccinated with 200cfu TAS2010 (◼) or BRD509 (○). A) Representative spleens at week 2 post-vaccination. B) Spleen weight was measured at the indicated time points post-vaccination, mean ± SEM shown. Data are pooled from 2–4 independent experiments (n = 9–20), at the indicated time points post-vaccination, where Wk 0 denotes data from naïve mice. Two-way ANOVA with Bonferroni post-tests were used for statistical analysis. (TIF) [file ppat.1011666.s002.tif]

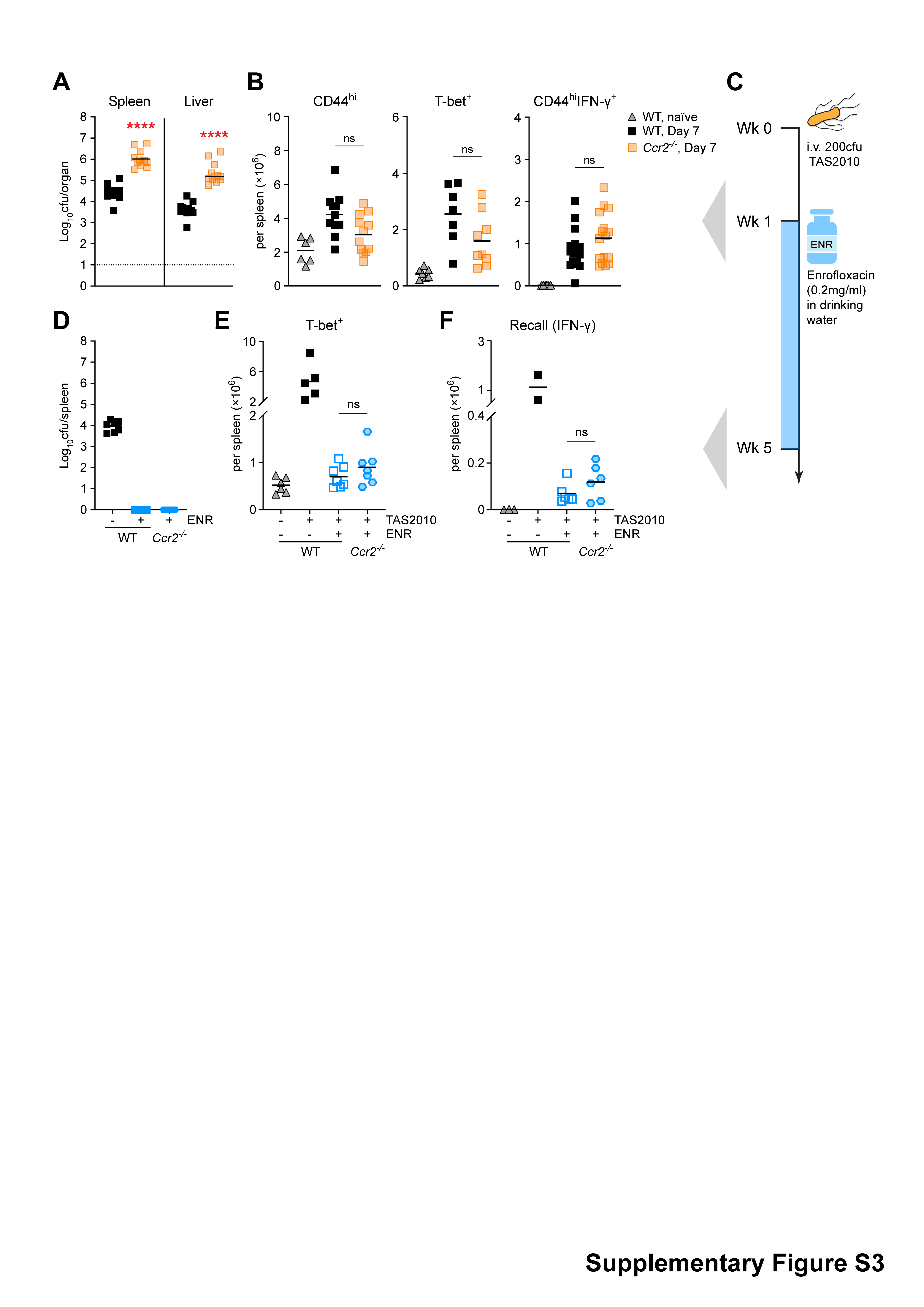

Supplement: S3 Fig — Wild-type C57BL/6 (black) or Ccr2-/- (orange) mice were either naïve or i.v. injected with 200cfu TAS2010. A, B) A) The bacterial load in the spleen and liver (geometric mean shown) and B) the number of activated CD4+ T cell subsets in the spleen were quantified at day 7 post-infection. IFN-γ secretion was measured using a diabody-based, ex vivo IFN-γ secretion assay. C-F) Mice were treated with 0.2mg/ml enrofloxacin (ENR) in the drinking water from Wk 1 until analysis at Wk 5 post-infection, as shown in C) the schematic diagram. D) Treated mice cleared bacteria in the spleen (geometric mean shown). The number of splenic CD4+ T cells that E) stained positive for T-bet intracellularly or F) produced IFN-γ following 18 hr of ex vivo re-stimulation with HKSTm was quantified. Data from individual mice are shown as symbols with group mean, pooled from 2–3 independent experiments. One-way ANOVA with Bonferroni’s post-tests were used for statistical analyses. (TIF) [file ppat.1011666.s003.tif]

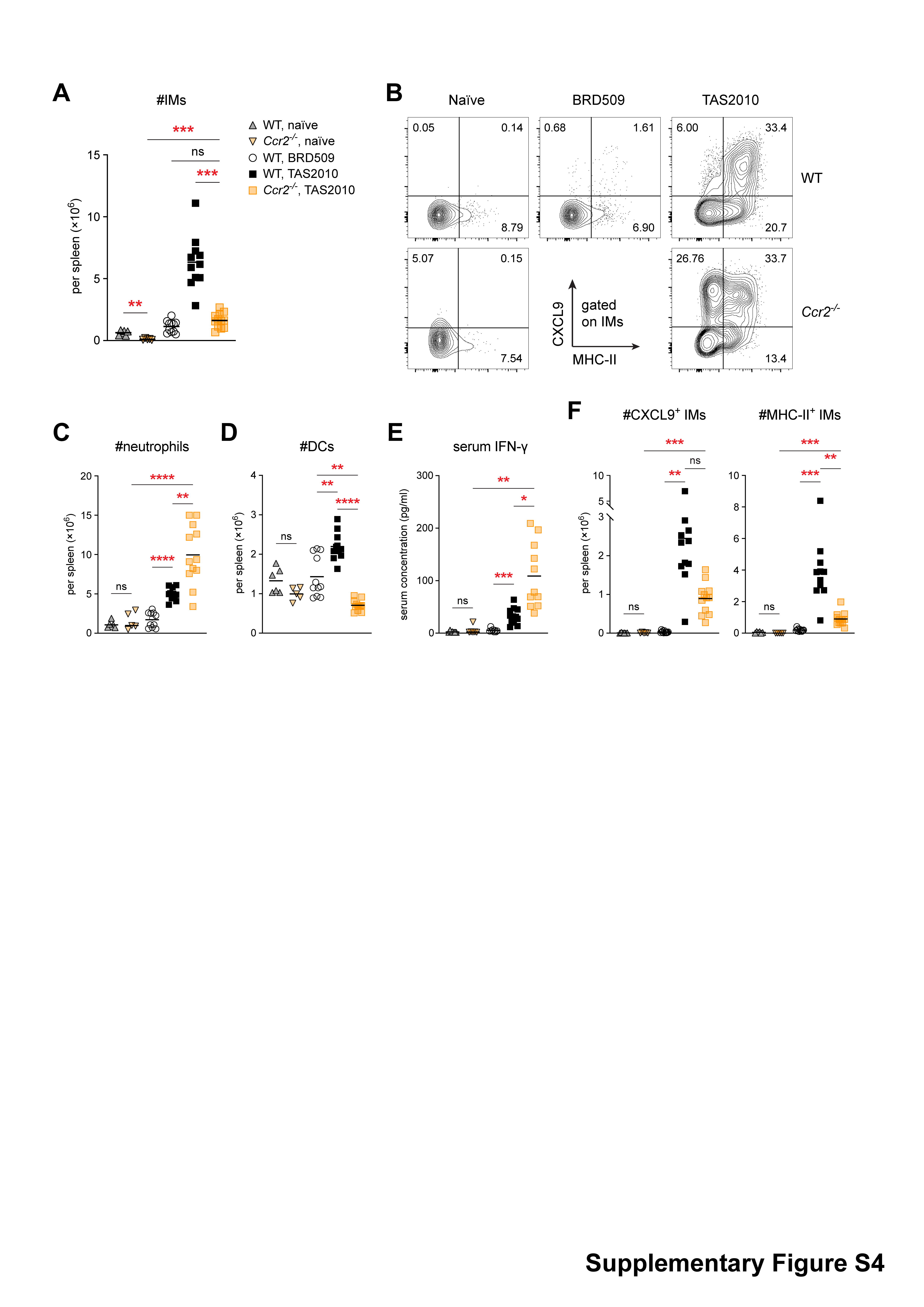

Supplement: S4 Fig — Wild-type C57BL/6 (black) or Ccr2-/- (orange) mice were either naïve or i.v. injected with 200cfu TAS2010 or BRD509. Mice were analysed at day 7 post-infection. A, C-D) The number of A) CD11b+Ly6GnegLy6Chi inflammatory monocytes (IMs), C) CD11b+Ly6G+ neutrophils and D) CD11c+MHC-IIhi conventional DCs were quantified. B, F) IMs were stained for MHC-II (surface) and CXCL9 (intracellular) and analysed by flow cytometry. B) Representative staining profiles are shown. F) the number of MHC-II- or CXCL9-expressing IMs was quantified. E) Serum concentration of IFN-γ was determined using the cytometric bead array (CBA). Data from individual mice are shown as symbols with group mean, pooled from 3–4 independent experiments. Brown-Forsythe and Welch ANOVA tests with Dunnett T3 corrections (a variation of One-way ANOVA that does not assuming equal variance) were used for statistical analyses. (TIF) [file ppat.1011666.s004.tif]

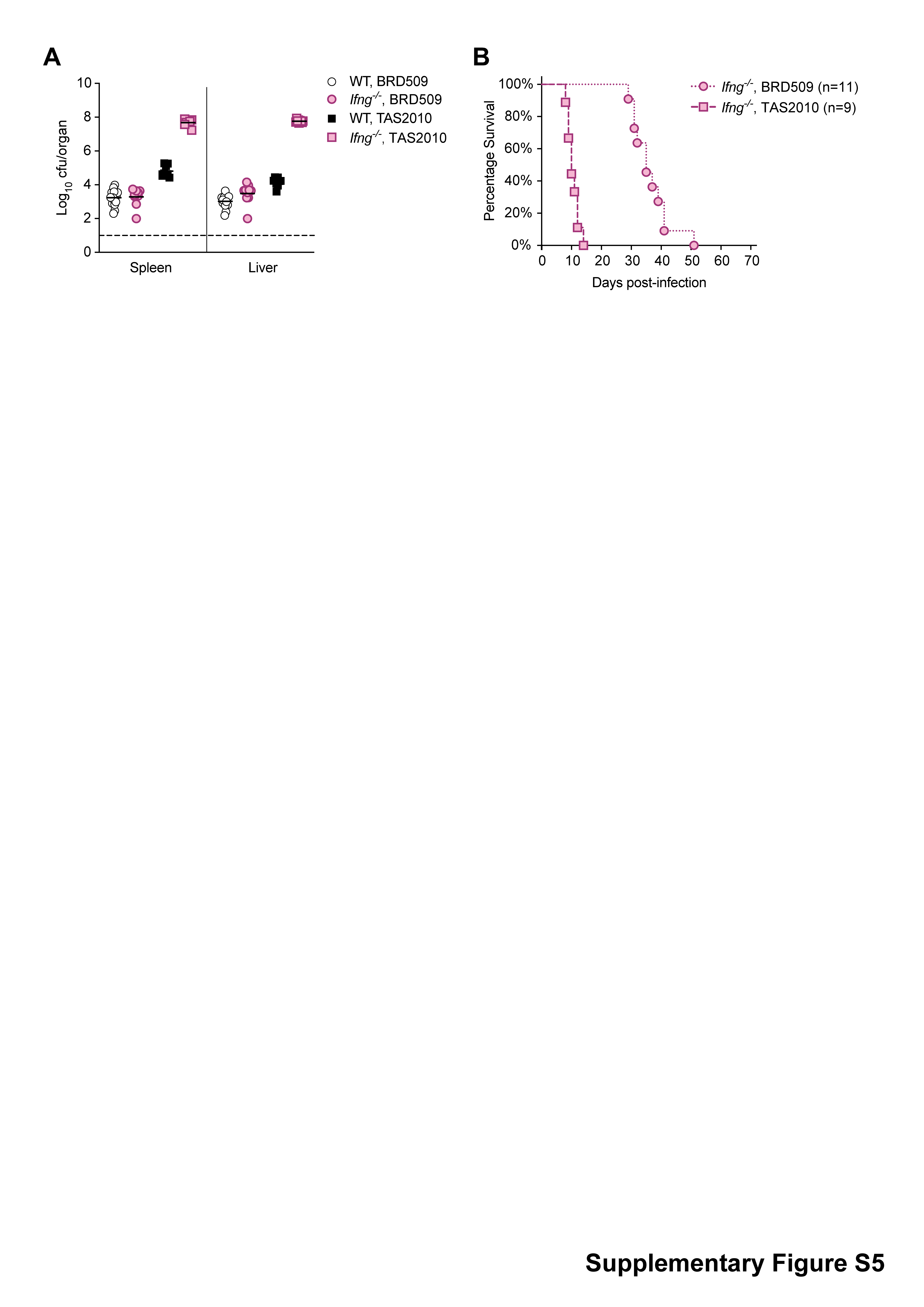

Supplement: S5 Fig — Wild-type C57BL/6 (black) or Ifng-/- (pink) mice were i.v. injected with 200cfu TAS2010 (square) or BRD509 (circle). A) The bacterial load in the spleen and liver from individual mice is shown with geometric mean for each group at day 7 post-infection. Data are pooled from 2 independent experiments. Two-way ANOVA with Bonferroni’s post-tests were used twice: first for comparing the two mouse genotypes, then for comparing the same genotype of mice infected with different S. Typhimurium strains. B) Shown is the percentage of mice remaining that were not considered moribund at the indicated time points post-infection. Data are pooled from 2 independent experiments. Log-rank Mantel-Cox test was used for statistical analysis. (TIF) [file ppat.1011666.s005.tif]
